# Planar Super‐Oscillation Diffractive Lenses: Practical Developments and Applications

**DOI:** 10.1002/nap2.70247

**Published:** 2026-08-14

**Authors:** Yutian Cheng, Lingyi Tang, Xingcheng Xiao, Xiaochang Yu, Ya Guo, Yiting Yu, Wenli Li

**Affiliations:** ^1^ Key Laboratory of Advanced Process Control for Light Industry (Ministry of Education) Jiangnan University Wuxi China; ^2^ Key Laboratory of Scale Manufacturing Technologies for High‐Performance MEMS Chips of Zhejiang Province, Key Laboratory of Optical Microsystems and Application Technologies of Ningbo City Ningbo Institute of Northwestern Polytechnical University Ningbo China; ^3^ Zhejiang Key Laboratory of Advanced Optical Functional Materials and Devices Ningbo University Ningbo China; ^4^ Ministry of Education Key Laboratory of Micro/Nano Systems for Aerospace, Key Laboratory of Micro/Nano Systems for Aerospace, Key Laboratory of Micro‐ and Nano‐Electro‐Mechanical Systems of Shaanxi Province School of Mechanical Engineering Northwestern Polytechnical University Xi'an China

**Keywords:** planar super‐oscillatory diffractive lenses, potential applications, practical multiple scenarios, super‐oscillatory lenses, supercritical lenses

## Abstract

As a type of inverse‐designed micro/nanophotonic device, planar super‐oscillation diffractive lenses (PSODLs) require complex trade‐offs among multiple key performance metrics to optimize their performance, including spot size, focusing efficiency, field of view (FOV), depth of focus (DOF), and working bandwidth. This review focuses on two representative types of PSODLs, super‐oscillatory lenses (SOLs) and supercritical lenses (SCLs). Both can generate far‐field focal spots with sizes below the diffraction limit, but they exhibit distinct optical‐field focusing characteristics, and there are trade‐offs among performance parameters. Therefore, this review provides specific guidance for the selection and optimization of PSODLs in accordance with application scenarios. To better promote the practical application of these kinds of lenses, a systematic analysis of typical applications in areas such as super‐resolution imaging, micro/nanofabrication, and high‐precision measurement is provided. Potential extensions of PSODLs to integrated optical imaging systems, optical sensing, particle manipulation, and optical storage are also discussed. Future practical deployment of PSODLs will rely on application‐oriented multiobjective optimization, robust and scalable fabrication, active system integration, and standardized performance evaluation.

## Graphical abstract

This review examines planar super‐oscillation diffractive lenses as a unified framework for super‐oscillatory lenses and supercritical lenses. It focuses on applications in super‐resolution imaging, micro/nanofabrication, and precision measurement and relates their application‐specific requirements to focal‐field control, multiobjective design strategies, and practical balances among resolution, focusing efficiency, sidelobes, working bandwidth, field of view, depth of focus, and manufacturability.

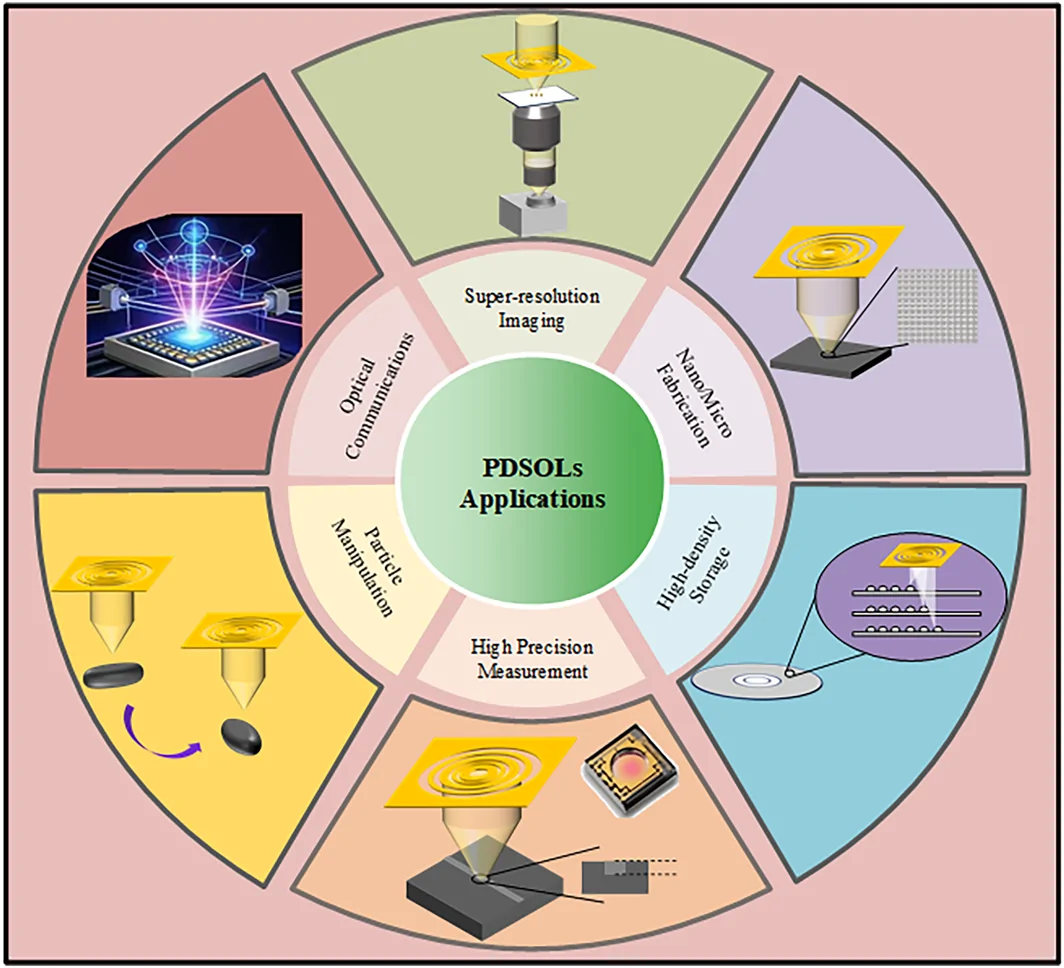

## 1 Introduction

With the development of micro/nanomanufacturing and computational design methods, planar diffractive lenses (PDLs) have received considerable attention in recent years [1, 2, 3, 4, 5, 6, 7, 8, 9]. Compared with their refractive counterparts, the most remarkable advantage of PDLs is their high degree of freedom for manipulating focal fields by optimizing micro/nanostructures to meet specific requirements. Planar super‐oscillation diffractive lenses (PSODLs) can also be classified as either super‐oscillatory lenses (SOLs) or supercritical lenses (SCLs), depending on whether the full width at half maximum (FWHM) is less than 0.38 λ/NA, where λ is the wavelength, and NA is the numerical aperture (NA = *n*sin*α*_max_, where *α*_max_ denotes the maximum convergence angle between the outermost ray and the optical axis of the lens) [10, 11]. SOLs have attracted significant attention due to their ability to defy the diffraction limit in the far field. The FWHM of the central focal peak can then be a deep subwavelength, which is less than 0.38 λ/NA. For practical applications, a balance between the FWHM of the central focal spot and the sidelobes is achieved in SCLs [12, 13, 14, 15], which exhibit a focal spot between 0.38 λ/NA and 0.50 λ/NA.

Super‐oscillation describes band‐limited functions containing local oscillations that are faster than those of the fastest Fourier components [16, 17, 18]. Originally introduced by Aharonov and coworkers in the context of quantum weak measurements [19], the principle was later extended to optics by Michael Berry [20, 21, 22, 23, 24, 25, 26, 27, 28, 29]. Berry and Popescu demonstrated that optical super‐oscillation could be achieved via subwavelength grating diffraction [21]. In 2007, Huang et al. fabricated a Penrose‐like quasiperiodic nanohole array on a metal film and experimentally observed subdiffraction optical super‐oscillatory hot spots at a distance of about 16 μm from the film, well beyond the near‐field region [30]. In 2009, Berry et al. further showed that super‐oscillation is not merely a mathematical construction but can arise naturally in monochromatic wave fields and complex optical fields [22]. In the same year, Huang and Zheludev proposed the concept of far‐field super‐resolution without evanescent waves, providing a new route toward label‐free and noninvasive optical super‐resolution [31]. In 2012, Rogers et al. reported a microscope based on SOLs, advancing super‐oscillation from the generation of localized hot spots to far‐field subdiffraction imaging [32]. More recently, planar SOLs composed of microstructures and nanostructures have benefited from rapid advances in nanofabrication and inverse design. These devices can be flexibly engineered to provide functionalities such as ultrahigh NA, extended depth of focus (DOF), and subdiffraction‐limit focusing in the far field.

However, such focal‐spot compression is typically accompanied by pronounced sidelobes, which can introduce background interference. SCLs offer a more balanced compromise between focal‐spot size and sidelobe suppression. In 2017, Qin et al. demonstrated an SCL optical label‐free microscope, showing far‐field subdiffraction imaging with an ultralong working distance [13]. Compared with conventional planar diffractive lenses, PSODLs provide greater flexibility in engineering far‐field subdiffraction optical fields. As illustrated in Figure 1, PSODLs demonstrate significant potential in applications including far‐field super‐resolution focusing and imaging [33, 34, 35, 36], high‐density data storage [37, 38, 39], nanolithography [40, 41, 42, 43, 44], precision measurement [45, 46, 47], and particle manipulation [48, 49, 50].

**FIGURE 1:**
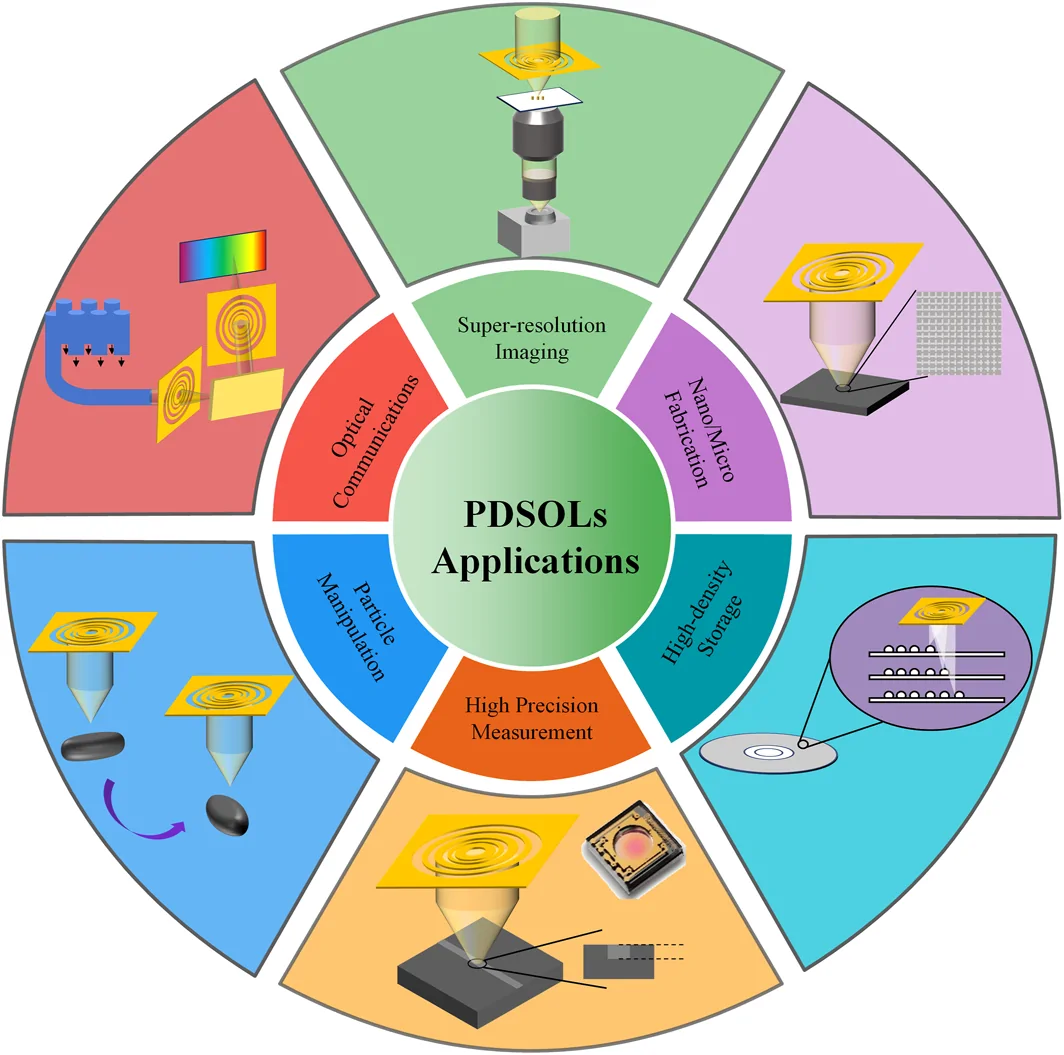
Application domains of PSODLs.

Super‐oscillation effects and their implementation in planar optical devices provide promising pathways for surpassing the conventional diffraction limit and acquiring super‐resolution information in the far field. PSODLs leverage subwavelength focusing capabilities and system integration advantages, demonstrating immense potential for transforming conventional optical systems, both theoretically and at the laboratory level. However, existing devices face significant trade‐offs among their working bandwidth, field of view (FOV), sidelobe suppression, focusing efficiency, and manufacturing repeatability, which hinder their transition from proof‐of‐concept to practical application. To enable laboratory prototypes to evolve into field‐deployable solutions, several requirements must be addressed: achieving broadband and achromatic operation, improving energy efficiency by sidelobe suppression, expanding the FOV, and developing cost‐effective manufacturing processes compatible with existing optical platforms. Therefore, a systematic review of current progress and design strategies across these key domains is essential.

Previous reviews have examined optical super‐oscillation from complementary perspectives, including subwavelength focusing and super‐resolution imaging, broader classes of PDLs, mathematical realization methods, and emerging super‐oscillation technologies [51, 52, 53, 54, 55]. Against this background, this review places SOLs and SCLs within a unified PSODL framework, with emphasis on application requirements and practical deployment. We intend to offer a practical, application‐oriented reference for researchers seeking to apply PSODLs by helping them translate the requirements of different application scenarios into appropriate design targets and performance trade‐offs. This review first examines the performance trade‐offs inherent in subdiffraction focusing and the corresponding multiobjective optimization methods. After discussing the performance trade‐offs and multiobjective optimization methods for subdiffraction focusing, it systematically analyzes the working principles of both scanning and nonscanning imaging modes in typical PSODL imaging applications. This review explores potential applications in the fields of micro/nanofabrication and high‐precision measurement, demonstrating how flexible light‐field customization can meet the requirements of these diverse scenarios. Finally, this review discusses key challenges and future directions for the practical deployment of PSODLs from the perspectives of fundamental performance trade‐offs, application‐driven design, manufacturability and system reliability, and standardized evaluation.

## 2 Design Considerations and Implementation Methods

### 2.1 Essential Performance Parameters for Practical Applications

Although planar SOLs can, in principle, produce arbitrarily small focal spots, this capability is governed by significant physical constraints and inherent performance trade‐offs [56, 57]. These constraints mainly appear as three interrelated characteristics: high‐intensity sidelobes, low main‐lobe efficiency, and a limited DOF. Consequently, the design of high‐performance PSODLs is fundamentally an inverse engineering problem [58]. It requires an optimal balance among key metrics, including DOF, NA, wavelength, FWHM, FOV, and focusing efficiency, within a multidimensional parameter space to meet specific target requirements [59, 60, 61, 62]. The key performance parameters are shown in Figure 2a. In Figure 2b, the radar chart presents normalized qualitative scores rather than universal quantitative limits. The assigned values illustrate the relative design priorities of representative lens types and the trade‐offs among these parameters in metalens design [63, 64, 65, 66].

**FIGURE 2:**
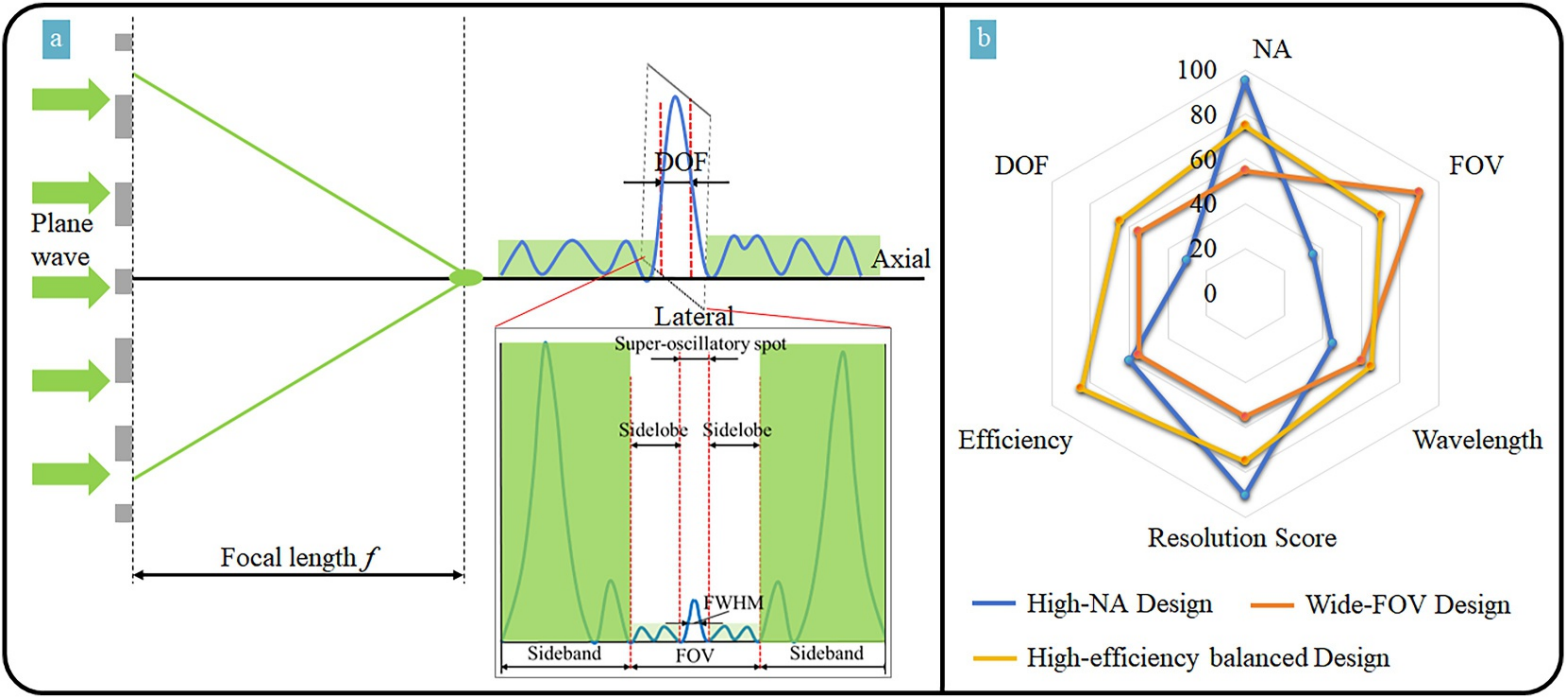
A schematic illustration of design trade‐offs. (a) Focusing characteristics of a planar super‐oscillatory diffractive lens. (b) Comparison of performance trade‐offs among representative design strategies.

In PSODLs’ design, the pursuit of higher resolution typically transfers more energy from the main lobe to the sidelobes. This reduces focusing efficiency and may introduce background noise, thereby degrading image contrast. Highly localized focal spots are also often accompanied by a reduced axial extent, namely, a shortened DOF, which limits three‐dimensional (3D) imaging and the characterization of thick samples. Furthermore, complex nanostructures designed for superior performance are more sensitive to manufacturing errors. Robustness optimization is often introduced in practical designs to balance device manufacturability and performance consistency. This relationship is summarized by the performance data in Figure 3a,b. Rather than simply listing the literature in chronological order, representative studies are categorized in Figure 3a,b, according to the primary functional objective and incident‐field condition of the devices, as detailed in the caption. Within each category, the reported FWHM, NA, operating wavelength, focusing efficiency, focal‐field profile, and fabrication method are compiled to compare the performance trade‐offs of different PSODL designs.

FIGURE 3Summary of the focusing performance of PSODLs. (a) (I–IV) Focusing on linearly and circularly polarized waves: (I) Reproduced with permission [67]. Copyright 2010, AIP Publishing. (II) Reproduced with permission [32]. Copyright 2012, Macmillan Publishers Limited. (III) Reproduced under the terms of the CC‐BY license [68]. Copyright 2016, the authors, Springer Nature. (IV) Reproduced with permission [69]. Copyright 2023, Elsevier Ltd. (V–VI) Focusing on cylindrically polarized vector waves: (V) Reproduced under the terms of the CC‐BY license [70]. Copyright 2016, the authors, Springer Nature. (VI) Reproduced under the terms of the CC‐BY license [71]. Copyright 2016, the authors, Springer Nature. (VII–IX) Broadband and achromatic: (VII) Reproduced under the terms of the CC‐BY license [72]. Copyright 2017, the authors, Springer Nature. (VIII) Reproduced under the terms of the CC‐BY license [73]. Copyright 2024, the authors, Institute of Optics and Electronics, Chinese Academy of Sciences. (IX) Reproduced with permission [74]. Copyright 2025, Chinese Laser Press. (b) (I–IX) Subdiffraction optical needles and diffractionless beams: (I) Reproduced with permission [75]. Copyright 2013, AIP Publishing. (II) Reproduced under the terms of the CC‐BY license [76]. Copyright 2014, the authors, Springer Nature. (III) Reproduced under the terms of the CC‐BY license [77]. Copyright 2017, the authors, Springer Nature. (IV) Reproduced with permission [42]. Copyright 2017, Optical Society of America. (V) Reproduced with permission [78]. Copyright 2022, Chinese Laser Press. (VI) Reproduced with permission [79]. Copyright 2022, AIP Publishing. (VII) Reproduced under the terms of the CC‐BY license [15]. Copyright 2023, the authors, MDPI. (VIII) Reproduced with permission [80]. Copyright 2023, Elsevier B.V. (IX) Reproduced with permission [81]. Copyright 2024, Optica Publishing Group. (X) Generation of subdiffraction 3D hollow spots: (X) Reproduced with permission [82]. Copyright 2018, Optical Society of America. (XI–XIII) Optical quantum super‐oscillation: (XI) Reproduced under the terms of the CC‐BY license [83]. Copyright 2016, the authors, Springer Nature. (XII) Reproduced with permission [84]. Copyright 2022, Optica Publishing Group. (XIII) Reproduced with permission [85]. Copyright 2022, Elsevier B.V. EBL: electron‐beam lithography; FIB: focused ion beam; LDW: laser direct writing.

**Figure:**
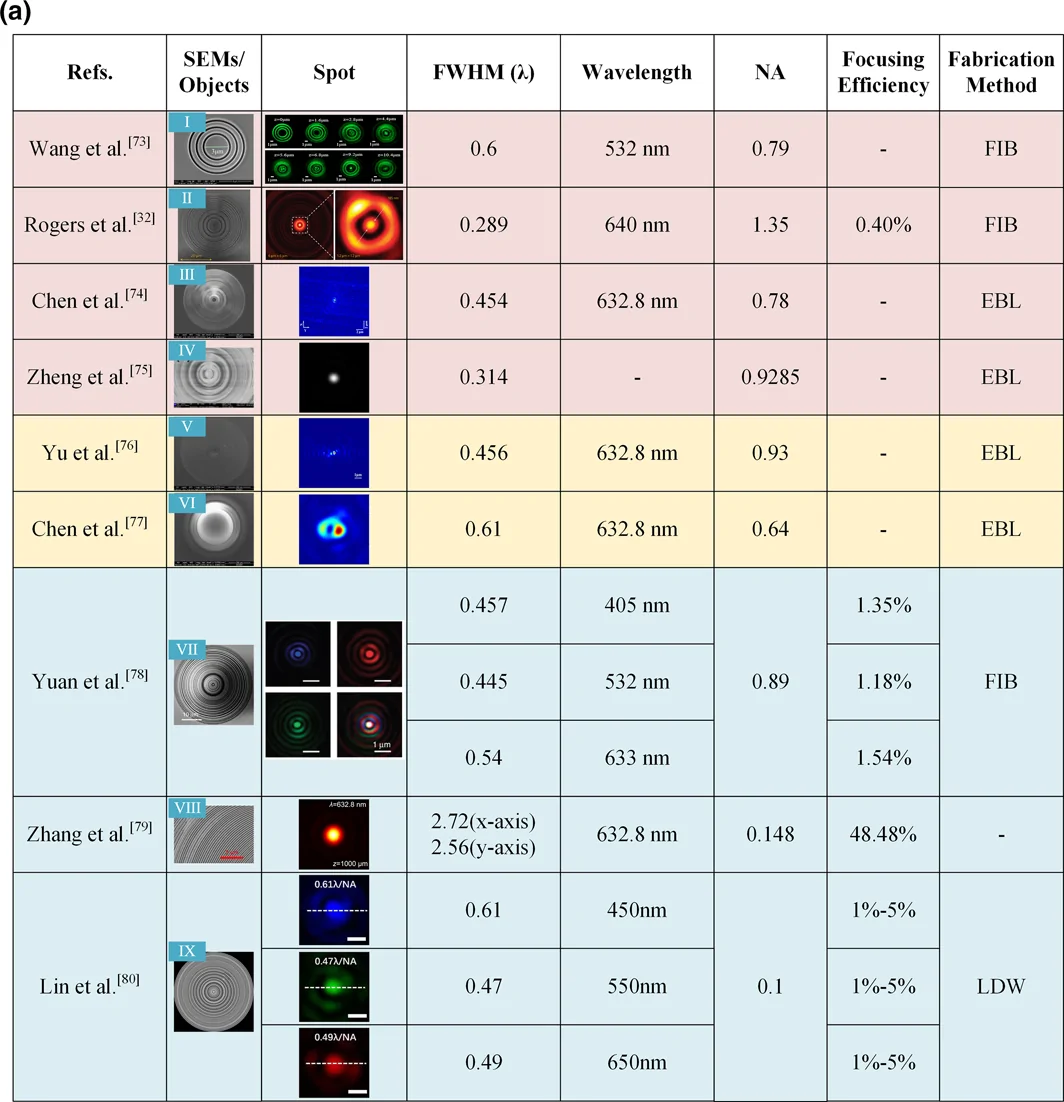

**Figure:**
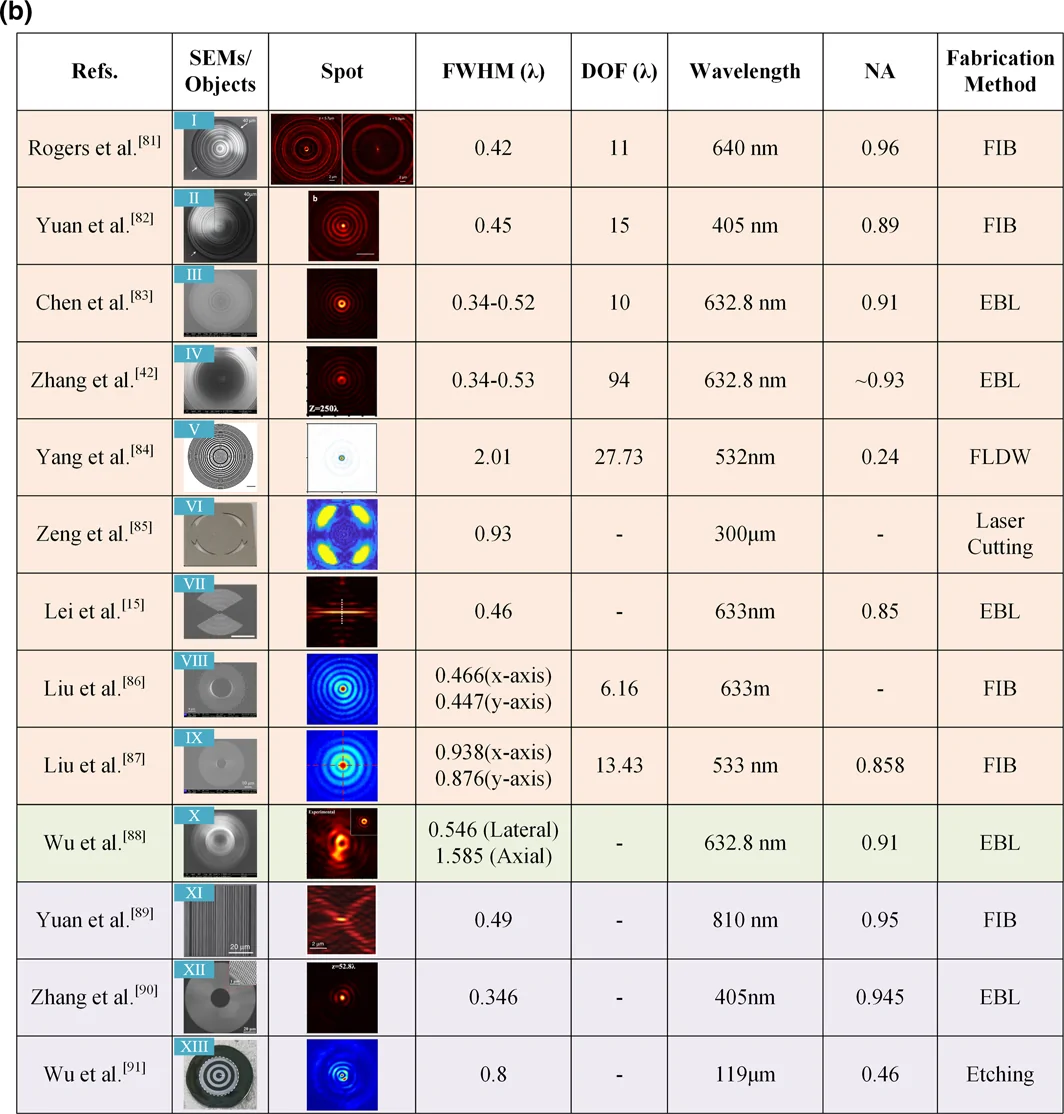

The compiled data in Figure 3a,b indicate that some designs prioritize resolution at the expense of focusing efficiency and DOF, whereas others relax the resolution requirements to obtain a longer DOF. This comparison highlights the need to prioritize performance indicators according to the requirements of a target application. For instance, resolution can be prioritized in intensity‐insensitive super‐resolution detection, whereas sidelobe suppression and focusing efficiency should receive greater emphasis in fluorescence imaging or optical manipulation. Future designs should make greater use of multiobjective optimization algorithms to search for constrained multidimensional design spaces and identify solutions that meet the requirements of specific applications in super‐resolution imaging and optical detection.

### 2.2 Inverse Design Methods for PSODLs

The design of PSODLs mainly relies on optimization algorithms. Among them, the particle swarm algorithm is widely used due to its simple implementation. Yan et al. used the particle swarm algorithm to optimize the design of a super‐oscillation lens that can convert linearly polarized light into a longitudinally polarized focal spot, achieving a focal‐spot size of 0.428 λ and a line width of 0.316 λ for super‐resolution imaging [86]. However, while dealing with the high‐dimensional parameter space of subwavelength structures, the particle swarm algorithm is prone to premature convergence to local optima and also faces challenges in simultaneously optimizing conflicting objectives such as diffraction efficiency, field‐of‐view angle, and spot size. To address these multiobjective design challenges, computational optimization methods such as genetic algorithms (GAs) have been widely adopted [87, 88, 89]. The traditional GA‐based design process is illustrated in Figure 4a. During standard GA iteration, the algorithm selects high‐performing solutions while maintaining population diversity through direct constraints. In practical applications, this approach often requires a large initial population and suffers from slow convergence. Furthermore, if constraints are strictly violated, the optimization efficiency can decrease significantly. To overcome these issues, we propose incorporating constraints into the objective function via a penalty function. This allows the multiobjective, multiconstraint problem to be transformed into an unconstrained multiobjective problem, enabling a more efficient iterative process (Figure 4b). However, phase values generated through random initialization and cross‐mutation are theoretically continuous. In practice, the fabrication of multistep SOLs is limited by material properties and processing techniques, which typically support only a specific number of discrete phase levels. To bridge this gap, we introduce a constraint function that effectively maps continuous phase values onto specified discrete steps during the generation process. This modified algorithmic flow is shown in Figure 4c.

**FIGURE 4:**
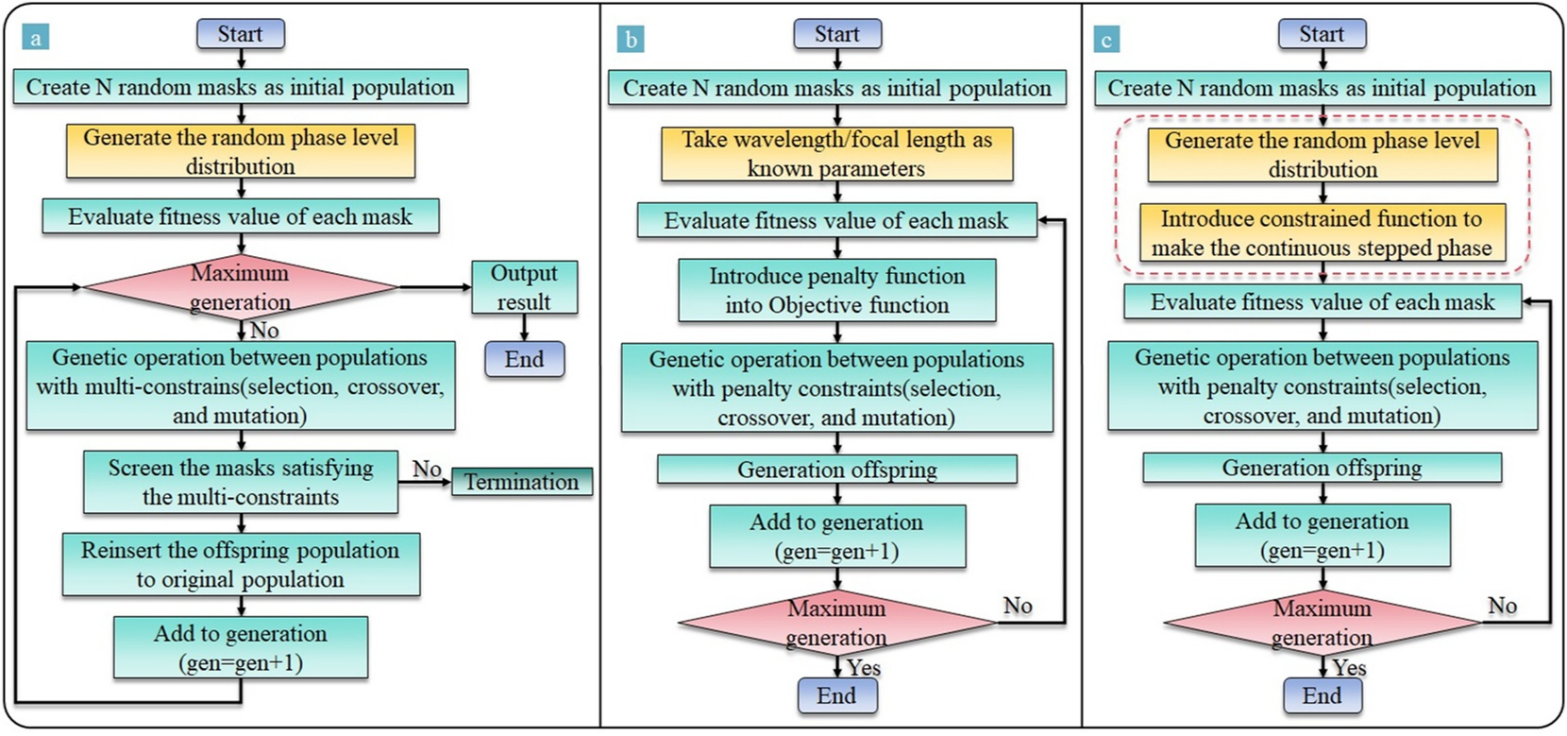
Genetic algorithm flowchart. (a) Traditional genetic algorithm. (b) Genetic algorithm with an introduced penalty function. (c) Genetic algorithm with an introduced constraint function.

With the advancement of optimization‐driven design methods, recent studies have introduced computational frameworks to address existing physical limitations. In 2024, Chen et al. proposed super‐oscillatory diffractive neural networks (SODNNs), which generate 3D optical super‐oscillations for super‐resolution imaging beyond the diffraction limit, as shown in Figure 5a [90]. SODNNs are constructed from diffractive layers that implement optical interconnections, whereas imaging samples or biological sensors provide the necessary nonlinearity. Conventional super‐oscillatory lens design methods typically optimize a two‐dimensional (2D) super‐oscillatory spot, which is often accompanied by significant sidelobes around the focal point, as shown in Figure 5b. In contrast, an SODNN modulates the incident optical field to produce optical super‐oscillation effects in 3D space, generating super‐resolved focal spots or optical super‐oscillatory needles, as illustrated in Figure 5c. In conventional photonic neural networks, diffraction‐limit resolution remains because these systems are generally trained without incorporating super‐oscillatory effects. By constructing large‐scale SODNNs that optimize the optical coefficients of stacked diffractive layers, it is possible to generate super‐oscillation in localized regions without sidelobes across the FOV. This methodology enables high‐performance super‐resolution imaging characterized by long working distances, extended DOF, and achromatic focal spots [91].

**FIGURE 5:**
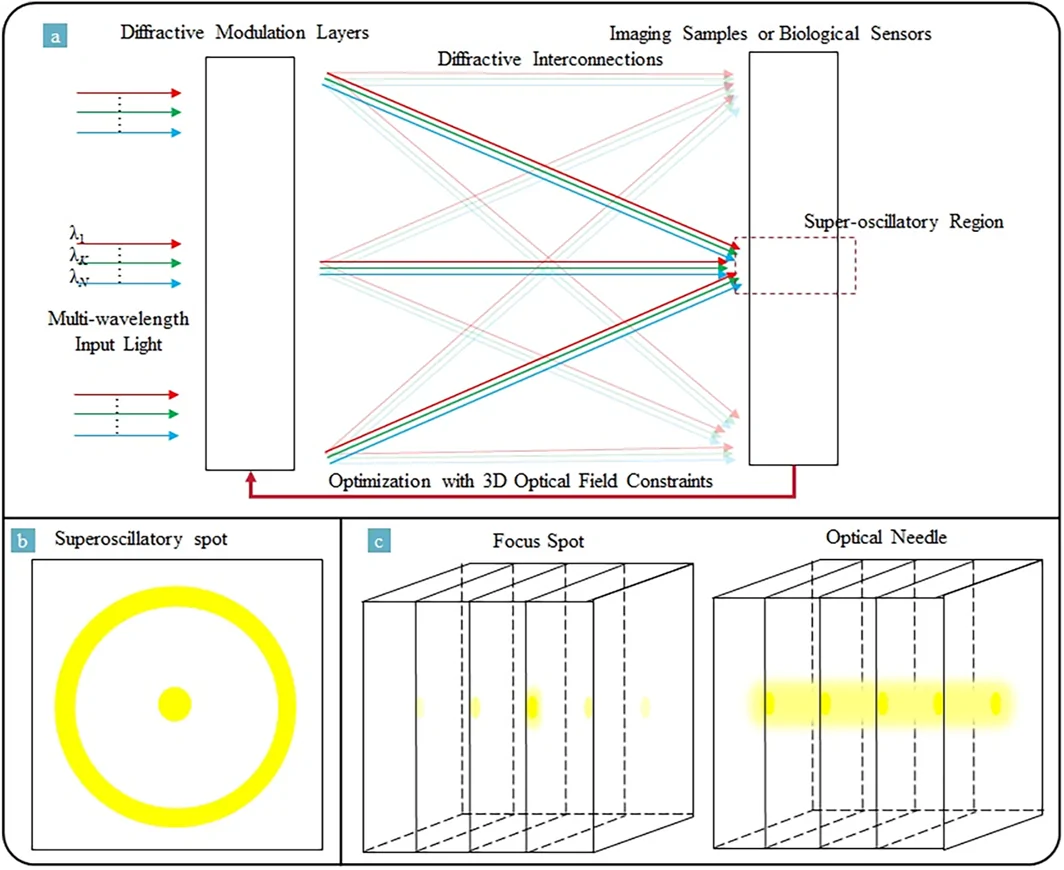
Training SODNNs to optimize the diffractive elements with 3D optical‐field constraints. (a) Utilizing diffractive modulation layers and free‐space propagation to implement the weighted optical interconnections, and imaging samples or biological sensors to implement nonlinearity. SODNNs can modulate the multiwavelength incident optical field to create optical super‐oscillation effects in 3D super‐oscillatory regions. (b) Conventional methods optimize a 2D focal spot at a specific focusing distance to achieve optical super‐oscillation with a large sidelobe. (c) Enlarged 3D super‐oscillatory regions show that SODNNs optimize the 3D optical field over a certain distance range to achieve super‐oscillation without the sidelobe.

The design methodologies and performance trade‐offs discussed in this chapter, particularly multiobjective optimization, provide the necessary framework to address these ongoing challenges. Furthermore, established research in light‐field modulation serves as a foundation for other domains requiring precise energy manipulation, such as micro/nanofabrication. This capability also underpins significant advances in high‐precision measurement. Specifically, the inverse design strategies and performance‐balancing principles fundamental to PSODL development enable the generation and sculpting of subdiffraction optical features. Such control is essential for noncontact, super‐resolution metrology, which is crucial for semiconductor inspection, materials characterization, and nanoscale quality control.

### 2.3 Flexible Optical‐Field Customization

#### 2.3.1 Adjustable Focusing Efficiency

In addition to generating long optical needles along the optical axis, PSODLs can also produce multifocal optical distributions in the longitudinal field, offering potential for the fabrication of ultraprecise and complex micro/nanostructures [92, 93, 94, 95]. This application requires not only resolution beyond the diffraction limit but also precise control over the number, spatial distribution, and energy allocation of focal spots. These requirements are well matched to the light‐field customization capability of SOLs. Specifically, inverse design in PSODLs provides an effective route for addressing the classical challenge of controlling energy distributions in multifocus systems. In 2017, Li et al. established a multiobjective optimization framework based on vector angular spectrum theory and a genetic algorithm [96]. This approach realized a simultaneous and programmable design of the number, position, size, and energy ratio of focal spots. It was used to generate 2–5 axial subdiffraction‐limit focal spots in a controllable manner, as shown in Figure 6a. A key contribution of this work was the ability to preset arbitrary intensity ratios among the foci, including nonuniform distributions such as 0.4:0.6:0.8:1, as shown in Figure 6b. By enabling flexible control while suppressing background noise and sidelobes, this strategy reduces cross talk among multiple foci and produces a cleaner focal‐field distribution. This work confirms the controllability of PSODLs for multifocal energy management and supports their use in micro/nanofabrication, particularly in multiregion parallel direct‐write lithography requiring differentiated exposure.

**FIGURE 6:**
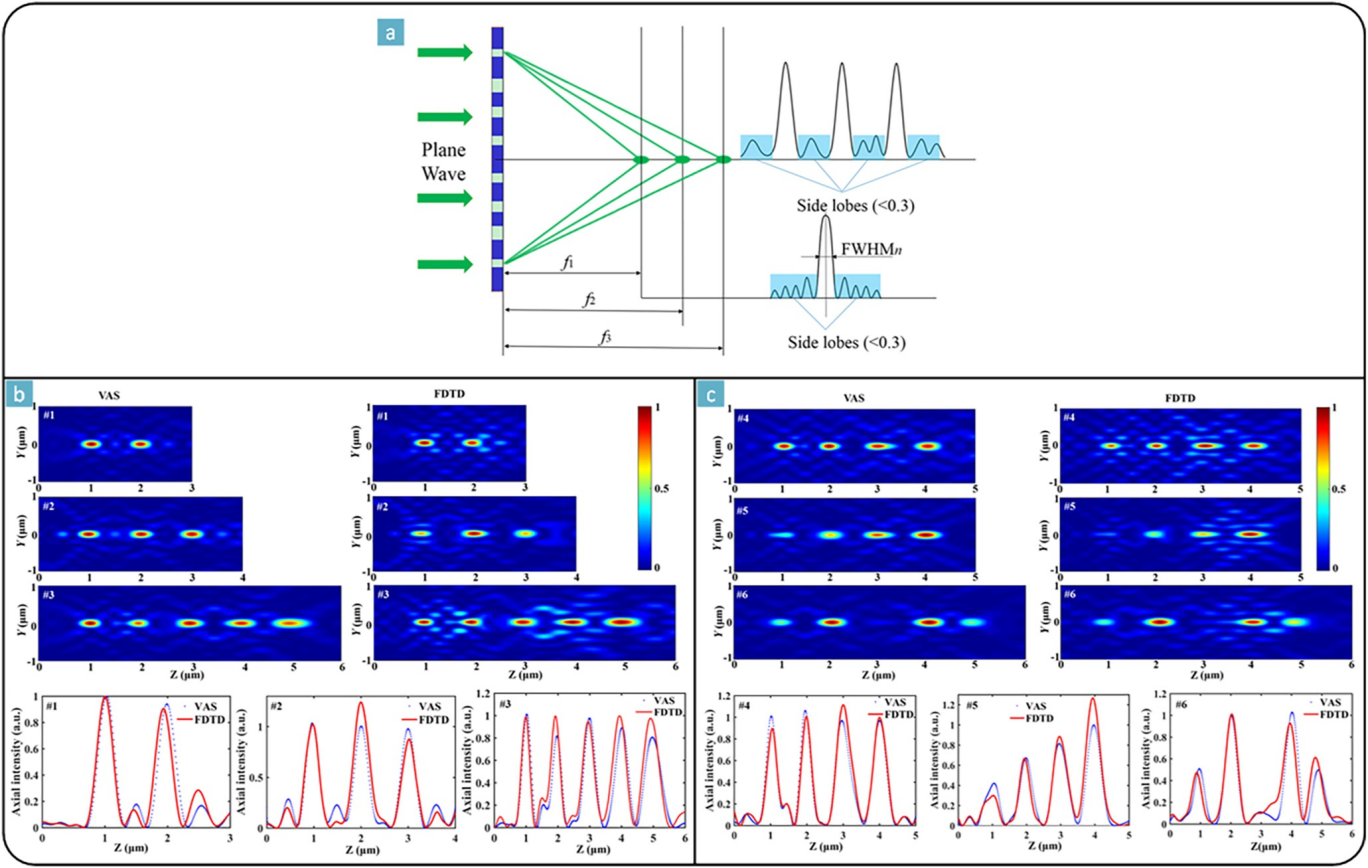
Design and performance of SOLs with multiple subdiffraction‐limit foci. (a) Schematic illustration of the optical modulation strategy to generate customizable multifocal light fields. (b) Comparison of the axial intensity distributions calculated using vectorial angular spectrum theory and the FDTD method, demonstrating SOLs capable of generating two to five focal spots with uniform intensity. (c) Axial intensity distributions of SOLs with preset nonuniform focal‐spot intensity ratios, demonstrating the precise control capability over the energy allocation among foci. Reproduced under the terms of the CC‐BY license [96]. Copyright 2017, the authors, Springer Nature.

#### 2.3.2 Radially Adjustable Light Fields

The FOV for PSODLs is critical for practical applications, such as nanoimaging, high‐density data storage, and ultrafine lithography. However, it is often limited by high‐energy sidelobes surrounding super‐oscillatory regions. In the case of PSODLs, manipulating the radial light‐field distribution requires balancing core performance metrics, including subdiffraction‐limit spot size, sidelobe intensity, and a limited FOV. Rogers et al. provided a quantitative foundation for this trade‐off by simultaneously optimizing the spot size and its intensity relative to the sidelobes over different FOVs [97]. Their work showed that radial‐field tuning first requires a deliberate choice of sidelobe level, spot size, and FOV, leading to two basic control strategies: passively suppressing sidelobes through scanning and filtering or directly enlarging the FOV by designing a sidelobe ring with a larger diameter. An alternative and more advanced approach, first demonstrated by Wu et al., treats sidelobes as a designable resource [82]. In this approach, a binary‐phase lens is designed to precisely manipulate the phase and polarization of sidelobes so that they form a bright ring surrounding a subdiffraction‐limit dark spot. The generation of subdiffraction 2D hollow focal spots is particularly important for stimulated emission depletion (STED) microscopy [98, 99] and nonlinear nanolithography [100].

The multifocal distribution of longitudinal optical fields can further expand the usable FOV of PSODLs. The aforementioned wavefront‐control strategy for the transverse profile of the focal plane is based on redistributing spatial‐frequency components and directing sideband energy. Extending this concept from a 2D focal plane to 3D propagation enables axial multifocal constraints and nondiffracting conditions to be incorporated into PSODL design, thereby transforming super‐oscillatory subwavelength compression from a single transverse profile into a longitudinal light‐field structure. Recent studies have investigated customized, functional, diffraction‐free light fields. For example, in 2022, He et al. designed an SOL that directly generated a static, large‐FOV light sheet with a subdiffraction‐limit thickness [101]. Using discrete focal‐spot synthesis, this strategy achieved a propagation length of 150.4λ, as shown in Figure 7a. This result extended radial‐field control from the 2D focal plane to 3D propagation space. The significance of this result lies in extending radial‐field control from a 2D focal plane to a 3D propagation space. By manipulating the axial distribution of radial energy, this device achieved both a large DOF and a large FOV, providing a synergistic scanning‐free illumination solution for 3D imaging. Other technical solutions focus on suppressing sidelobes through systematic codesign with main‐lobe optimization. In 2023, the self‐trade‐off optimization method proposed by He et al. was used to successfully fabricate ultrathin light sheets with excellent sidelobe suppression characteristics [102], as illustrated in Figure 7c. This approach balances key metrics, such as light‐sheet thickness, nondiffraction length, and sidelobe level, achieving a subdiffraction‐limit thickness, quasi‐nondiffraction propagation, and a low sidelobe ratio (SR) of 15.4%. The resulting window‐like energy distribution also exhibited a theoretical SR as low as 7.6%, providing a promising illumination scheme for super‐resolution fluorescence microscopy of microfluidic chips requiring extremely high background suppression.

**FIGURE 7:**
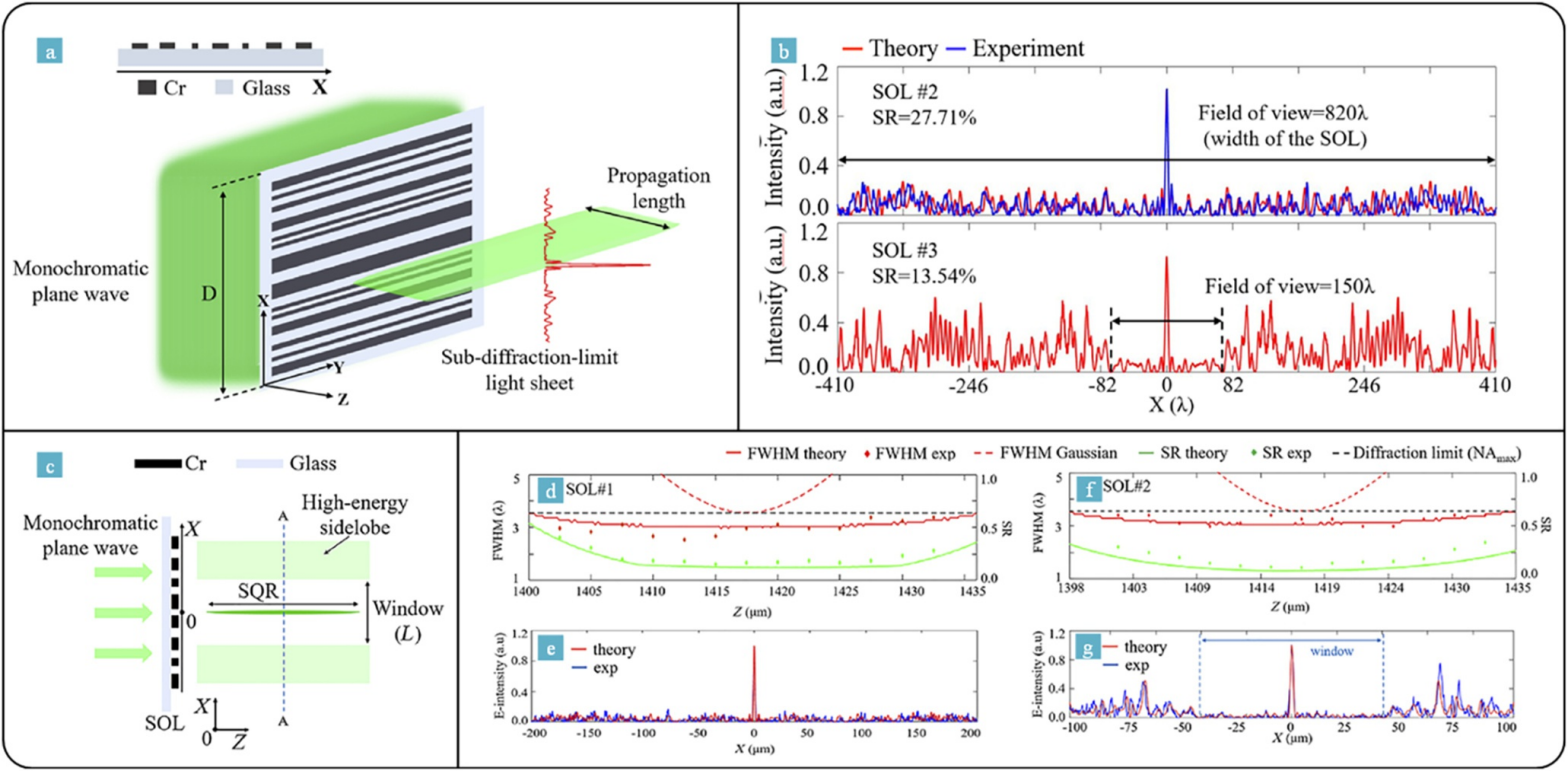
Engineered super‐oscillatory lenses for advanced light‐sheet generation. (a, b) Enlarging the FOV and DOF simultaneously. (a) Schematic diagram of an SOL generating a subdiffraction‐limit light sheet. (b) Comparison of normalized intensity profiles for SOLs with a FOV of 150λ and 820λ, demonstrating the capability to tailor the lateral coverage while maintaining a subdiffraction thickness. Reproduced with permission [101]. Copyright 2022, Optica Publishing Group. (c–g) Actively suppressing sidelobes within a tailored light sheet. (c) Schematic diagram of a sidelobe‐suppressed subdiffraction‐limit quasi‐nondiffracting light sheet (SQLS), highlighting the optimization window concept. (d, e) Performance of a standard SQLS (SOL #1): (d) The FWHM remains below the diffraction limit across the subdiffraction‐limit quasi‐nondiffracting range, whereas the SR is inherent. (e) Corresponding intensity profile showing the SR. (f, g) Performance of an optimized SQLS with a confinement window (SOL #2): (f) Subdiffraction focusing is maintained. (g) The intensity profile reveals drastically suppressed sidelobes (SR ∼7.6%) within the target window, achieved by a novel self‐trade‐off optimization method that redistributes sidelobe energy outside the window. Reproduced with permission [102]. Copyright 2023, Optica Publishing Group.

#### 2.3.3 Dispersion‐Tunable PSODLs

Acquiring spectral information across a broad frequency band with high fidelity is one of the core challenges in modern optical measurements and imaging. Traditional spectroscopic techniques rely on bulky dispersive components, such as gratings and prisms, which make the systems complex and difficult to integrate. For PSODLs and related flat optical systems, inverse design and dispersion engineering allow dispersion to be treated both as an aberration to be suppressed and as a controllable design degree of freedom for optimizing broadband focusing, achromatic response, and multiwavelength field manipulation [103, 104].

This design perspective supports new routes toward realizing on‐chip integrated spectroscopic measurements. For example, Yuan et al. achieved subwavelength‐scale achromatic super‐oscillation focusing in the near‐infrared and visible‐light bands by using a binary amplitude and phase mask to control the wavefront [72]. The schematic diagram of the simulated focused pattern in the X–Z vertical section is shown in Figure 8a. Zhang et al. achieved super‐resolution focusing with an NA as high as 0.95 across multiple visible wavelengths by using wavefronts with a binary‐phase zone plate [107]. Lu et al. proposed and developed a monolayer metasurface based on a titanium dioxide nanopillar array, as shown in Figure 8b, which achieved subdiffraction focusing across the entire visible spectrum for the first time [105]. This work demonstrates that precise dispersion engineering with nanostructures can fully overcome the extreme wavelength sensitivity of super‐oscillation effects, enabling precise dispersion control over a broad bandwidth. Lu et al. further introduced a polarization multiplexing mechanism, which allowed the same metasurfaces to achieve diffraction‐limited focusing in a single polarization state and multiwavelength achromatic subdiffraction focusing in another [108].

**FIGURE 8:**
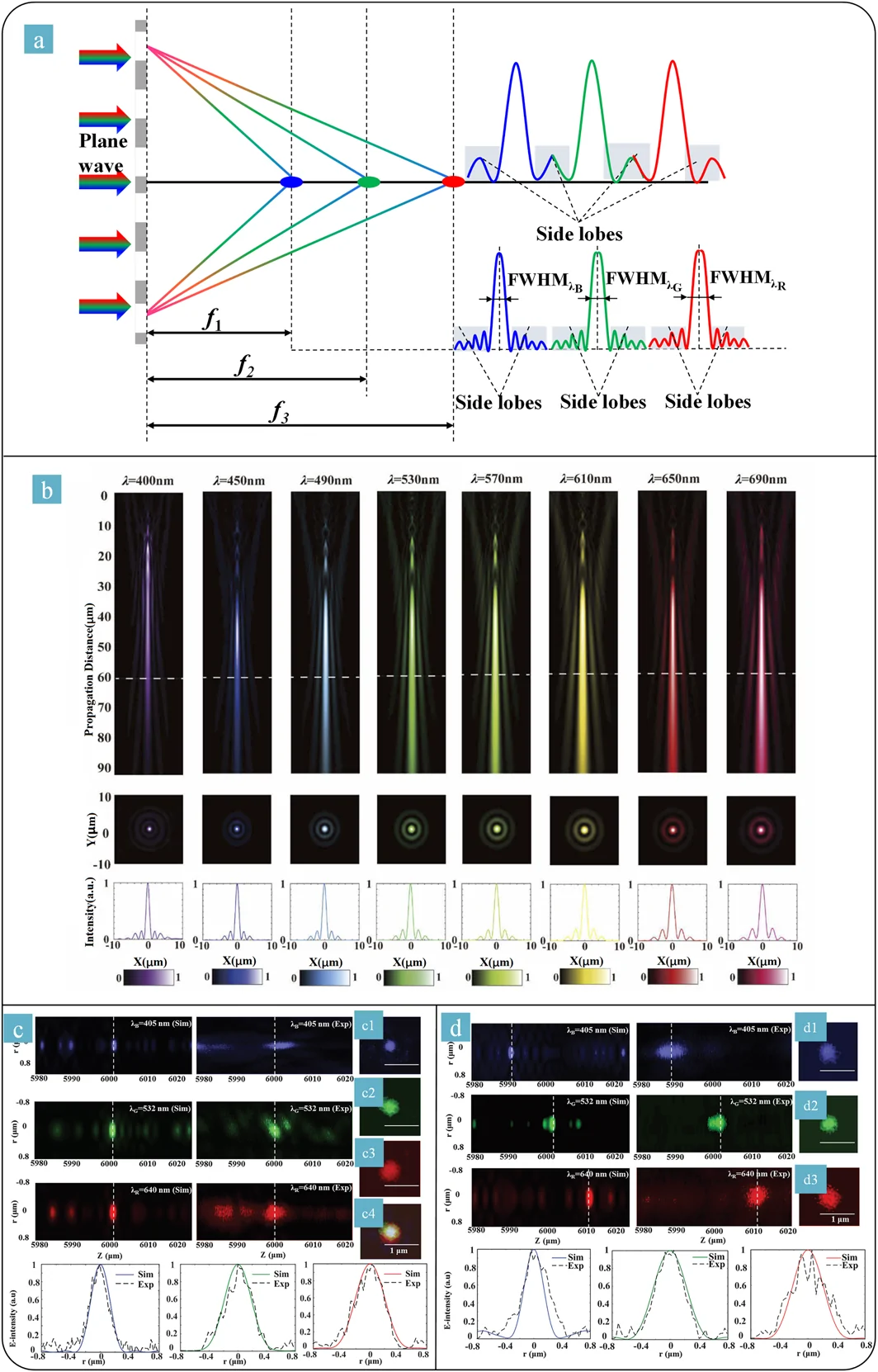
Planar achromatic super‐oscillatory lens. (a) Simulated focusing pattern in the X–Z cross‐section. (b) Light‐field distributions along the propagation direction (top). Intensity distributions at the preset focal plane position *z* = 60 μm (middle). Point‐spread function of the broadband achromatic super‐oscillatory metasurface at the preset focal plane position (bottom). Reproduced with permission [105]. Copyright 2021, Optical Society of America. Experimental focusing pattern in the X–Z cross‐section. The white vertical dashed lines indicate the focal planes. (c) Experimentally recorded intensity patterns in the transverse focal plane: (c1) for the blue wavelength (λB), (c2) for the green wavelength (λG), (c3) for the red wavelength (λR), and (c4) for the fused image from RGB wavelengths. (d) Simulated and experimental normalized intensity distributions in the transverse focal plane along the dashed lines through the focal points: (d1) for λB, (d2) for λG, and (d3) for λR. All images were captured by a built‐in color CCD camera in the microscope. Reproduced with permission [106]. Copyright 2019, The Royal Society of Chemistry.

Although multiple studies have focused on suppressing dispersion, our group previously explored flexible modulation and inverse design of dispersive elements, extending the design space beyond conventional achromatic correction [106]. We demonstrated the direct and effective manipulation of chromatic aberrations in SOLs by proposing a multiobjective, constraint‐free model for designing centimeter‐scale achromatic SOLs with micrometer‐scale feature sizes. Using this approach, centimeter‐scale achromatic SOLs were designed, as shown in Figure 8c. Furthermore, by employing a universal algorithm, the authors implemented an inverse design of dispersive properties, creating an achromatic‐customized SOL where the focal order by wavelength is reversed compared to standard diffractive lenses, as shown in Figure 8d. All foci maintained subdiffraction‐limit sizes at a working distance of approximately 11,000 λ, providing ample space for practical imaging. This result demonstrates that customized dispersion control in dielectric SOLs is not only crucial for achieving achromatic performance but also serves as a core functionality for constructing compact spectral imaging systems, offering a potential route to replace traditional bulky beam‐splitting devices. Together, these studies show that dispersion‐engineered PSODLs can extend beyond achromatic focusing to support broadband field control, wavelength‐dependent focal tailoring, and compact spectral imaging. Such capabilities provide a basis for integrating PSODLs into multifunctional measurement systems in which dispersion is treated as a design variable rather than only a limitation to be corrected.

## 3 PSODLs for Various Practical Applications

### 3.1 PSODLs for Super‐Resolution Imaging

PSODLs allow subdiffraction‐limit focusing in the far field, which can be directly applied to microscopy, data storage, and sensing. Imaging methods utilizing PSODLs can be categorized into scanning and nonscanning modes based on the included working principles [109, 110, 111, 112, 113, 114]. This section discusses the potential applications of PSODLs for label‐free far‐field imaging beyond the diffraction limit, which is one of the most promising applications for this type of lens.

#### 3.1.1 Scanning Imaging

The limited FOV, inevitable sidelobes, and micrometer‐scale working distances of PSODLs pose significant challenges for direct imaging applications [115]. To address these limitations, PSODL‐based confocal microscopy configurations have been developed. In these setups, a subdiffraction‐limit hot spot generated by PSODLs serves as a point‐like or needle‐like illumination source for point‐to‐point or line scanning [116, 117]. Specifically, PSODLs are positioned at the conjugate plane of the objective lens to deliver subdiffraction‐limit incident light onto a sample. A pinhole filter placed at the imaging plane of the confocal system then effectively suppresses high‐intensity sidelobes, thereby enhancing image contrast and resolution. The final image with subdiffraction‐limit resolution is reconstructed through raster scanning of the sample and sequential integration of the acquired intensity profiles.

The groundbreaking capability of this technology was validated through imaging of a metallic‐film nanohole array by Rogers et al. [32]. As shown in Figure 9a, two holes with a minimum spacing of 41 nm were nearly resolved [118]. This result unequivocally breaks the diffraction limit of traditional optical microscopy, marking the advent of PSODLs as a label‐free, purely optical, far‐field super‐resolution imaging tool. However, the limited DOF and working distance of the first‐generation SOLs constrained their ability to image complex structures. Subsequent research has focused on performance optimization, driving the transition from proof‐of‐principle demonstrations to practical applications. As shown in Figure 9b, Fei Qin et al. developed SCLs with long‐DOF characteristics, which could not only clearly reproduce all the sophisticated details of a nonperiodic “Lion Head” pattern but also perform complete imaging of complex 3D fishnet wedge structures [13]. What is particularly noteworthy is that this system successfully distinguished a 65‐nm gap at a wavelength of 405 nm. This value is not only well below the Rayleigh diffraction limit but also significantly surpasses the resolution limit of 0.38 λ/NA for conventional single‐mirror SOLs. This demonstrates their powerful potential for handling complex samples and further advancing SOLs from proof of concept toward practical imaging applications.

**FIGURE 9:**
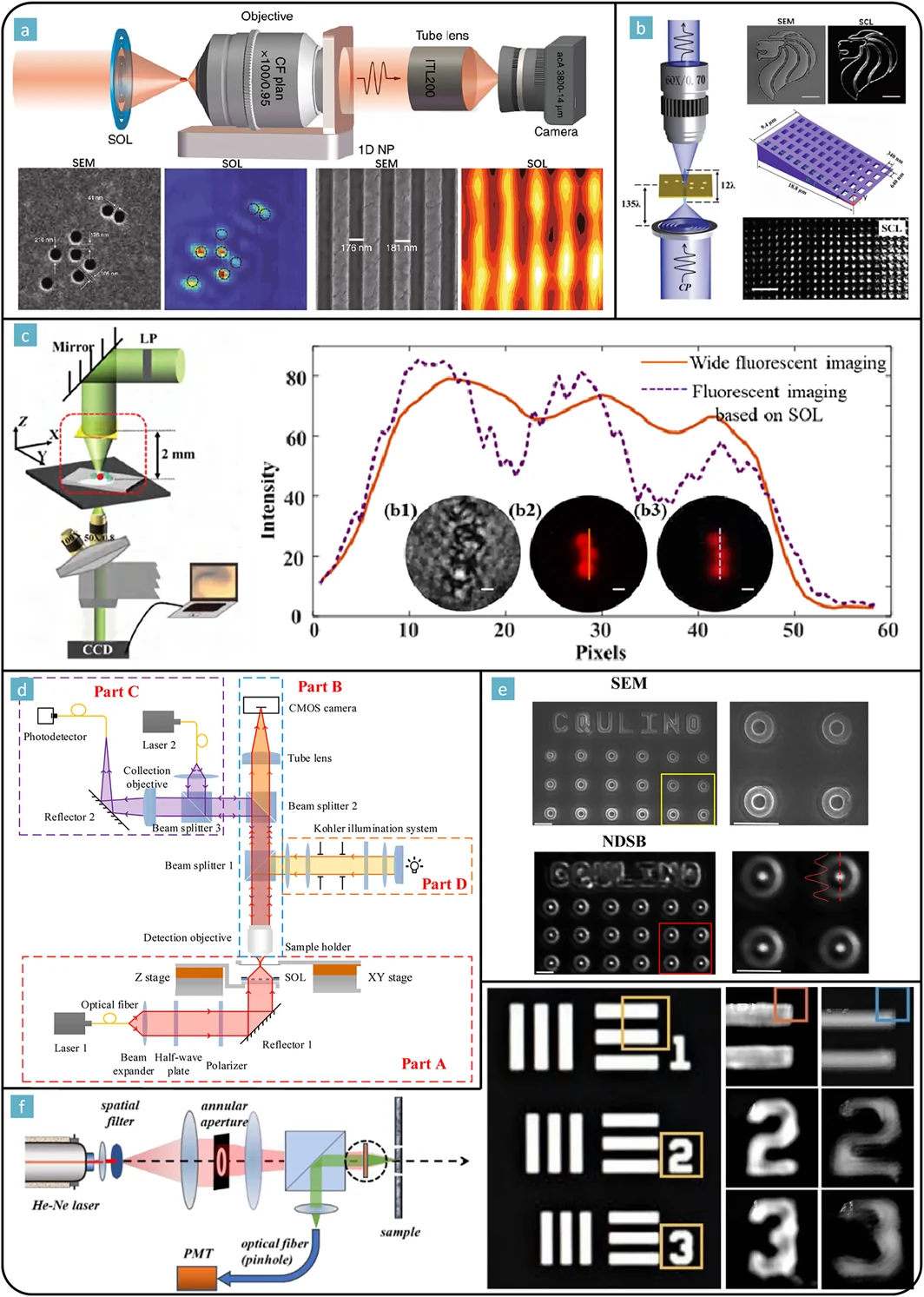
PSODL‐based scanning imaging microscopy. (a) Super‐oscillatory fields are obtained by a high‐NA microscope with an objective lens mounted on a 1D nanopositioner (1D NP); the camera captures the 2D intensity distribution of the diffraction pattern. Top to bottom: SEM image of a cluster of 210 nm nanoholes in a metal film. An SOL image of the nanohole cluster (dashed circles map the positions of the holes). An SEM image of a subdiffraction grating with a linewidth of approximately 180 nm. A diffractionless SOL image of the grating obtained in the confocal setup with a conventional microscope with NA = 0. Reproduced under the terms of the CC‐BY license [118]. Copyright 2019, the authors, Springer Nature. (b) Schematic diagram of SCL microscopy and imaging of a large‐scale nonperiodic pattern. Reproduced with permission [13]. Copyright 2017, Wiley‐VCH Verlag GmbH & Co. KGaA, Weinheim. (c) Schematic diagram of the imaging measurement setup for dispersive fluorescent particles. The imaging results of fluorescent nanoparticles obtained by employing bright‐field microscopy, wide‐field fluorescence microscopy, and SOL‐based microscopy, respectively. Scale bar, 1 μm. Reproduced with permission [119]. Copyright 2020, the authors, published by The Royal Society of Chemistry. (d) Schematic diagram of the overall optical path. Part A: Subdiffraction focusing and scanning module; Part B: Infinity imaging and virtual confocal detection module; Part C: Laser confocal positioning module; and Part D: Conventional epi‐illumination module. Reproduced with permission [120]. Copyright 2023, Elsevier B.V. (e) SEM image of the measured sample (top left); the zoomed‐in SEM image of the concentric circles (top right); the imaging results by NDSB microscopy (bottom left); the zoomed‐in image of the concentric circles by NDSB microscopy, with the solid red line displaying the corresponding intensity profile (bottom right). Scale bars: 1 μm. Reproduced with permission [121]. Copyright 2023, Elsevier B.V. (f) Schematic diagrams of the designed microscopy setup (red represents incident light, and green represents reflected light). Schematic diagram of the samples (resolution test target, left). Conventional CLSM imaging results of the samples (resolution test target, center). CSOL imaging results of the samples (resolution test target, right). Reproduced with permission [69]. Copyright 2023, Elsevier Ltd. LP, linear polarizer.

In parallel, the working distance and application scope of PSODLs have continued to expand. As shown in Figure 9a, Gang Chen et al. extended the working distance to several hundred micrometers by generating a nondiffracting super‐oscillatory beam, substantially enhancing the capability of SOLs for noncontact operation over large spatial scales [118]. More importantly, SOLs have been successfully applied to pinhole‐free fluorescence imaging. Our team achieved ultra‐resolution observation of sample morphology in the fluorescence mode [119]. At a wavelength of 532 nm, a resolution of approximately 1.07 λ was achieved, outperforming the Rayleigh diffraction limit and extending the utility of SOLs from scattering‐based imaging to the broader domain of biological fluorescence labeling (Figure 9c). Recent advancements include the super‐oscillatory microscope developed by Liu et al., which integrates laser confocal positioning functionality to achieve the precise alignment of the focal plane. It successfully resolved grating line widths of approximately 255 nm at a wavelength of 633 nm (Figure 9d) [120]. Additionally, Zhou et al. demonstrated label‐free super‐resolution microscopy using a nondiffracting super‐oscillation beam, achieving deep penetration and 3D imaging of complex patterns (Figure 9e) [121]. In addition, it achieved a gap resolution of 180 nm at a wavelength of 632.8 nm. Furthermore, Zheng et al. demonstrated a compound super‐oscillatory lens designed for reflective confocal imaging, achieving an experimental resolution of 1.12 μm and an SR below 2%, with significantly suppressed sidelobes [69]. Their study provides a schematic diagram of the microscopy setup alongside experimental confocal imaging results and intensity profiles for a resolution test target, as illustrated in Figure 9f. In 2025, Ye et al. designed a binary‐phase‐type planar super‐oscillation device, which can be used in combination with a zoom lens [122]. By extending super‐oscillation scanning imaging from the microscopic scale to the macroscopic scale, this work achieved a focal spot smaller than the Rayleigh diffraction limit at a working distance of 20 m. This work marks a step toward applying SOL technology to practical far‐field detection.

Conventional optical devices exhibit inherent trade‐offs among NA, DOF, chromatic aberration, and focal‐spot size. To address these coupled constraints, inverse design provides a useful route for balancing multiple performance metrics. Our team employed a multiobjective GA to design an advanced SOL that simultaneously provides an extended DOF and achromatic performance [123]. A schematic diagram of the system is shown in Figure 10a. Experiments demonstrated that this device could focus blue, green, and red light beams simultaneously into an optical needle with a diameter of ∼0.5 λ at a working distance of 428 μm. The extended DOF is particularly useful for 3D imaging. As shown in Figure 10b,c, the SOL microscope achieved high‐resolution imaging across the entire depth range in a single scan while successfully imaging both a complex 3D metallic wedge and neural tissue up to 170 μm thick. This approach eliminates the need for time‐consuming axial scanning required in conventional methods with limited DOF, thereby significantly enhancing the efficiency of 3D imaging.

**FIGURE 10:**
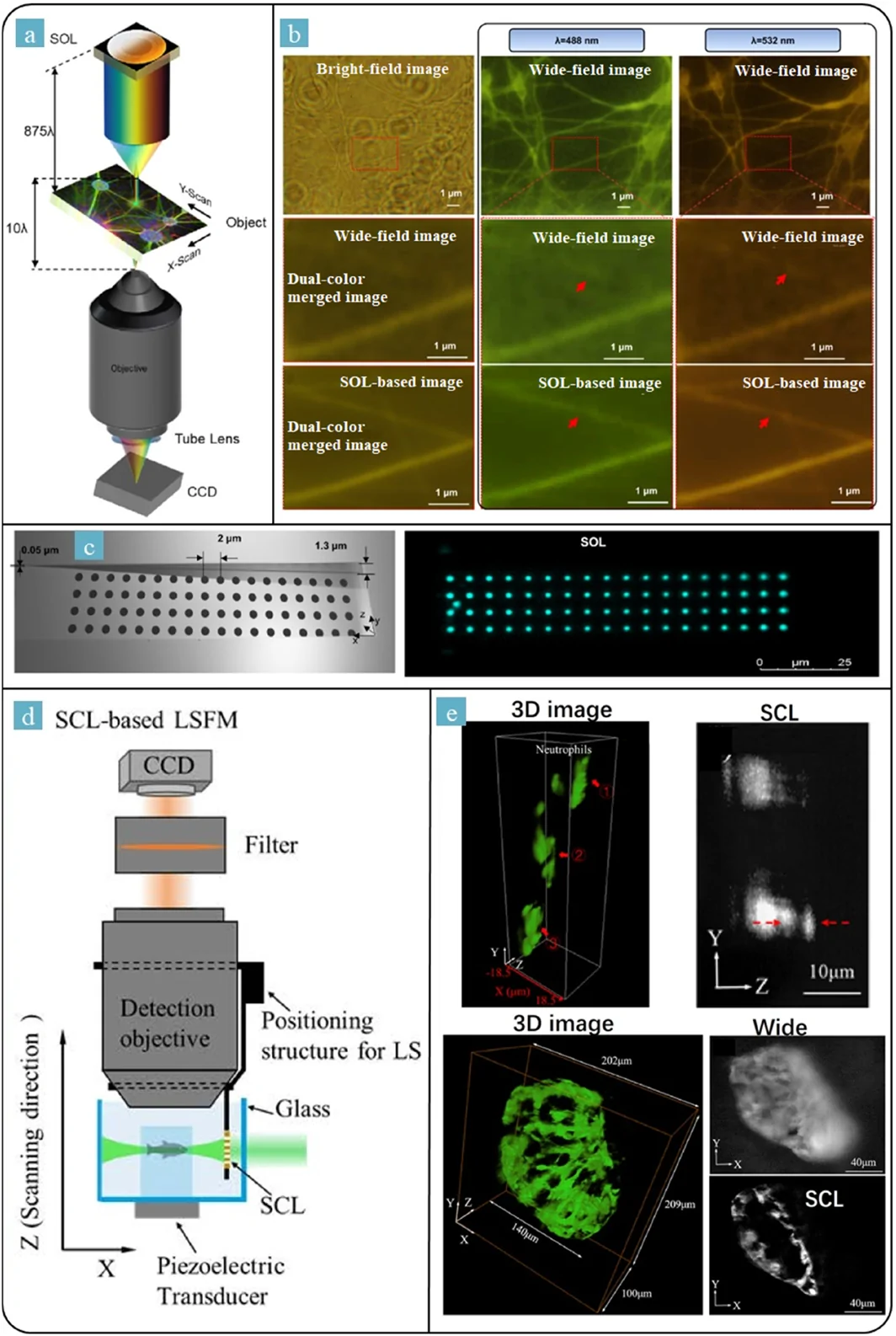
Multicolor subdiffraction imaging with an SOL‐based microscope. (a) Sketch of an SOL‐based scanning optical microscope. (b) Sketch of a 3D metallic fishnet wedge composed of an array of circular holes with a period of 2 μm. Images of the wedge collected by the wide‐field microscope, LSCM, and the DOF‐extended SOL‐based microscope, all operating in the transmission mode. (c) Bright‐field and wide‐field fluorescence microscopy images of thick neuron tissue. Zoomed‐in views of the enclosed areas. SOL‐based microscopy images of the enclosed area in merged dual‐color fluorescence images from wide‐field imaging and the SOL‐based microscope. The red arrows denote the fine structures of neurons. Reproduced under the terms of the CC‐BY license [123]. Copyright 2023, the authors, published by Springer Nature. (d) Schematic diagram of the optical path of the SCL‐based LSFM. (e) Schematic diagram of fluorescently labeled zebrafish larvae. Imaging results of neutrophils: 3D image and Y–Z sectional images at the positions indicated by Arrow 2 (*x* = 0 μm) (top). Three‐dimensional image of fluorescently labeled zebrafish larval hearts captured by SCL‐based LSFM. The X–Y projection images of the heart were obtained by wide‐field fluorescence imaging and SCL‐based LSFM, respectively (bottom). Reproduced with permission [124]. Copyright 2025, Optica Publishing Group.

The practical value of PSODLs can be further enhanced by integrating them with advanced imaging modalities. For instance, He et al. integrated an SCL‐based illumination module with light‐sheet fluorescence microscopy (LSFM) [124]. As shown in Figure 10d, a single planar SCL chip replaced the complex illumination lens assembly in a conventional LSFM system. This design not only significantly simplifies the system architecture but, more importantly, also enables the generation of a light sheet that simultaneously offers a large FOV and subdiffraction‐limit thickness. The imaging capability of this integrated system was validated using living samples. The SCL‐based LSFM successfully captured high‐resolution three‐dimensional images of fluorescently labeled organs, providing clear optical sectioning with better contrast than traditional wide‐field fluorescence imaging. Furthermore, the SCL‐generated light sheet maintains a large FOV and highly uniform axial resolution, overcoming the divergence and edge decay problems inherent in traditional Gaussian light sheets. Through the synergy of inverse design and system integration, SCLs can serve as customizable planar optical elements for high‐performance super‐resolution imaging.

#### 3.1.2 Nonscanning Imaging

Point‐to‐point scanning imaging substantially prolongs image acquisition, making PSODL‐based super‐resolution microscopy challenging to apply in practical scenarios requiring high speeds, such as live‐cell fluorescence imaging. To address this limitation, recent studies have shifted from scanning to nonscanning imaging strategies. Rather than using a single hot spot for point‐by‐point acquisition, these strategies employ multifocal spot arrays designed to illuminate different regions of the sample simultaneously [123, 124]. Early attempts validated the feasibility of synchronous multifocal imaging, laying a theoretical foundation for super‐resolution microscopy that operates without mechanical scanning and for real‐time imaging of dynamic processes. For instance, Valle et al. proposed a design for multifocal diffractive lenses based on Zernike polynomials [125]. The resulting lens could simultaneously image planes at different depths, providing a conceptual foundation for subsequent multiplane synchronous detection.

In contrast to the multifocal parallel‐scanning approach discussed above, another widely explored strategy is to modulate the transfer function of the imaging system directly. This strategy belongs to wide‐field nonscanning imaging and avoids physical pinhole scanning. By placing specially designed phase or amplitude masks at the spectral plane of the optical path, the system can capture, in a single exposure, the entire image convolved with a customized point‐spread function, thereby preserving the subdiffraction‐limit features of the target in wide‐field imaging. The first real‐time subdiffraction‐limit linear imaging system, with a far‐field working distance based on SOLs, was demonstrated as part of the optical super‐microscope (OSM) [125], as shown in Figure 11a. This design avoids fine scanning, data postprocessing, and sample pretreatment. This OSM distinguished two 15 μm‐diameter circular apertures separated by 55 μm (center‐to‐center). This design effectively reduced the focal‐spot width to 72% of the diffraction limit and improved the two‐point resolution to 75% of the Rayleigh criterion. By suppressing high‐energy sidelobes, the system mitigates interference, thereby enhancing image quality and the signal‐to‐noise ratio.

**FIGURE 11:**
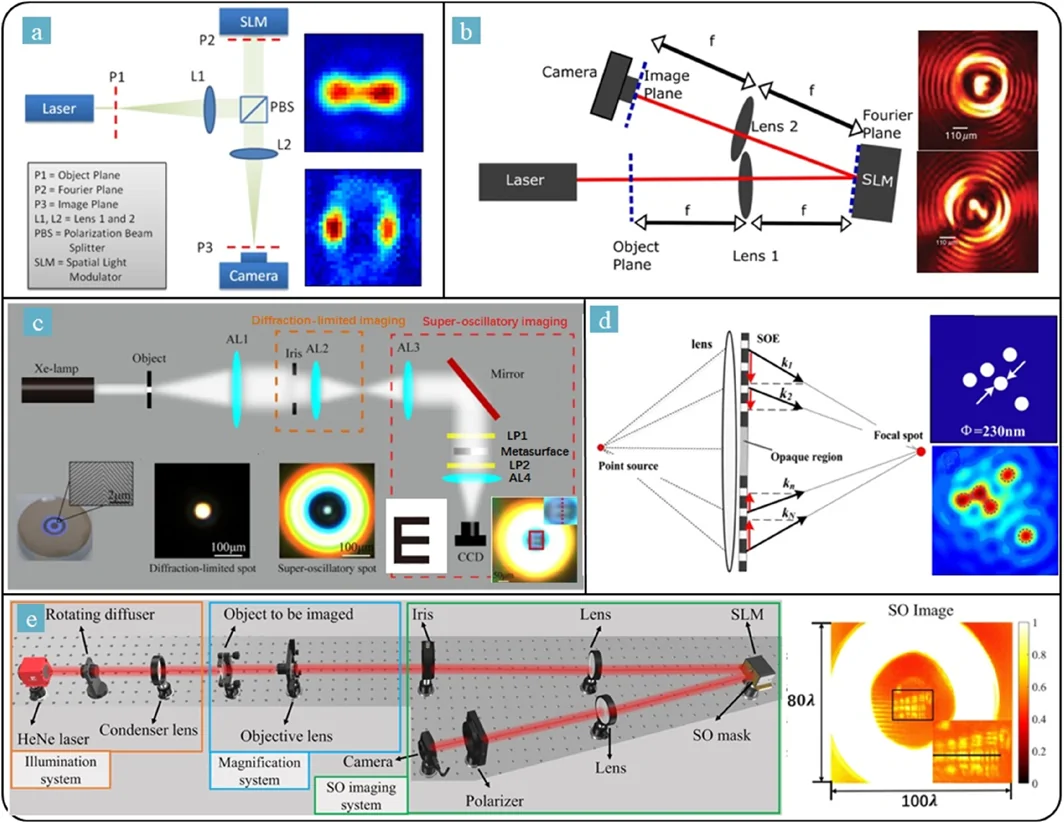
(a) Schematic diagram of the OSM. Reproduced with permission [125]. Copyright 2013, Springer Nature. (b) Schematic diagram of the experimental setup for super‐resolution far‐field imaging of complex objects using reduced super‐oscillatory ripples, including results for the letters “E” and “N.” Reproduced with permission [126]. Copyright 2017, Optical Society of America. (c) Broadband achromatic super‐oscillatory imaging using a super‐oscillatory pupil filter. Reproduced with permission [127]. Copyright 2018, Wiley‐VCH Verlag GmbH & Co. KGaA, Weinheim. (d) Formation of a super‐oscillatory waveform generated by hybrid phase‐amplitude super‐oscillatory elements and corresponding simulation results for sparse point‐source imaging. Reproduced with permission [128]. Copyright 2019, Optical Society of America. (e) Experimental imaging setup for single‐shot super‐oscillatory imaging (left) and high‐NA diffraction‐limited versus super‐oscillatory imaging results of a USAF resolution target (right). Reproduced under the terms of the CC‐BY license [129]. Copyright 2023, the authors, published by AIP Publishing.

Dong et al. proposed a new class of super‐oscillatory waveforms consisting of a subdiffraction‐limit hot spot and low‐energy sidelobe ripples, which served as a customized point‐spread function for a conventional 4F imaging system [126]. As shown in Figure 11b, super‐resolution imaging of the Latin letters “E” and “N” was achieved using a 4F imaging device incorporating optical coherence tomography (OCT). To expand the working bandwidth, Li et al. achieved broadband super‐resolution imaging at 0.64 times the Rayleigh criterion with visible light (400–700 nm) using a 4F imaging system, as shown in Figure 11c [127]. Additionally, to enhance the practicality and imaging quality of nonscanning imaging, Xie et al. proposed a hybrid phase‐amplitude super‐oscillatory element scheme for nonscanning optical super‐resolution imaging [128]. As shown in Figure 11d, algorithmic optimization enabled the system to distinguish point targets with a spacing of 247 nm during the imaging process. The resulting images showed clearer point‐source separation and higher contrast. In 2023, Yang et al. achieved single‐exposure super‐oscillation imaging of extended objects by significantly expanding the FOV using a generalized periodic mask [129]. The configuration of the experimental imaging system is illustrated in Figure 11e, together with the subdiffraction‐limited imaging results of USAF targets at high NA.

### 3.2 Fabrication of Micro/Nanostructures

PSODLs offer several advantages for high‐resolution direct writing lithography by generating subdiffraction‐limited focal spots. Additional flexible light‐field customization capabilities, such as the precise control of focusing efficiency and radial distribution, offer immense potential for fabricating complex three‐dimensional structures. Conventional laser direct writing (LDW) technology, based on the diffraction limit, faces resolution constraints governed by wavelength, making it increasingly difficult to meet the growing demands of nanoscale manufacturing. By generating localized subdiffraction‐limit hot spots, PSODLs provide a novel optical tool for the direct writing, etching, and modification of nanoscale features. Here, the use of PSODLs as high‐resolution light sources is explored for direct fabrication, followed by a discussion of how customizable light fields could drive intelligent and functionalized advanced manufacturing.

PSODLs demonstrate exceptional flexibility for light‐field manipulation, providing novel technical pathways for the direct fabrication of micro/nanostructures. Previous studies have investigated the high‐resolution fabrication of optical devices within the ultraviolet band. For example, Fu et al. proposed an aluminum nitride‐based SCL scheme [130]. Operating at a wavelength of 405 nm, this lens employed a binary‐phase modulation strategy, utilizing only AlN nanopillars with diameters of 60 and 150 nm as the basic units. This approach simultaneously achieved a subdiffraction‐limit focal spot, an extended DOF, and effective suppression of sidelobe interference, as shown in Figure 12a–c. Experimental results demonstrated that grating patterns fabricated by these SCLs in LDW processes exhibited significantly superior line resolution and contrast compared to those produced by traditional Fresnel zone plates (FZPs). The distinguishable grating spacing was reduced from approximately 540 to 480 nm, fully validating the outstanding potential of such meta‐atom modulation devices for ultraviolet micro/nanofabrication.

In the short‐wavelength deep‐ultraviolet band, Li et al. systematically compared phase‐modulated metalens arrays with amplitude‐modulated chromium film homogenizers, offering two complementary solutions for static and dynamic beam shaping [131]. Furthermore, Liu et al. designed a planar diffractive lens for the vacuum ultraviolet band, achieving sub‐100‐nm focal‐spot sizes while maintaining high transmission efficiency [132]. They also systematically analyzed aberrations in refractive lenses and their effects on focusing performance, offering new insights for super‐resolution imaging and material characterization in the vacuum ultraviolet band. For large‐area applications in the visible spectrum, achromatic SCLs recently developed by Lin et al. [74] provide an important example. This work moves beyond a conventional reliance on complex subwavelength meta‐atoms, instead adopting a multilevel diffractive lens architecture. This type of polymer lens was fabricated using grayscale laser lithography, exhibited a diameter of 10 mm, and comprised 1251 concentric rings. The device also achieved achromatic super‐diffraction focusing with a spot size of 0.88 times the Airy disk size across the entire visible spectrum (400–700 nm). The resulting centimeter‐scale aperture, polarization‐insensitive characteristics, and broadband achromatic performance enabled direct compatibility with conventional white‐light or supercontinuum laser sources, providing a viable technological foundation for the integration of large‐area, high‐throughput lithography and data storage systems.

In contrast to the techniques discussed above, which are based on meta‐atoms or multilevel diffraction, PSODLs rely on a diffractive physics‐based modulation principle, which introduces new possibilities for laser microfabrication. He et al. constructed a super‐oscillatory light field by managing high‐order diffraction, thereby eliminating any dependence on subwavelength resonant structures [133]. Using femtosecond laser pulses, the team created ablation areas beyond the Rayleigh criterion on a chromium film, achieving a direct writing processing resolution of 400 nm, as shown in Figure 12d–f. This result enabled the successful etching of nanoscale slits with widths below the diffraction limit and the fabrication of Fresnel zone plates with high structural fidelity. This achievement demonstrates that the precise fabrication of complex optical devices can be achieved solely through macroscopic control of the diffractive field, without employing nanoscale meta‐atoms.

**FIGURE 12:**
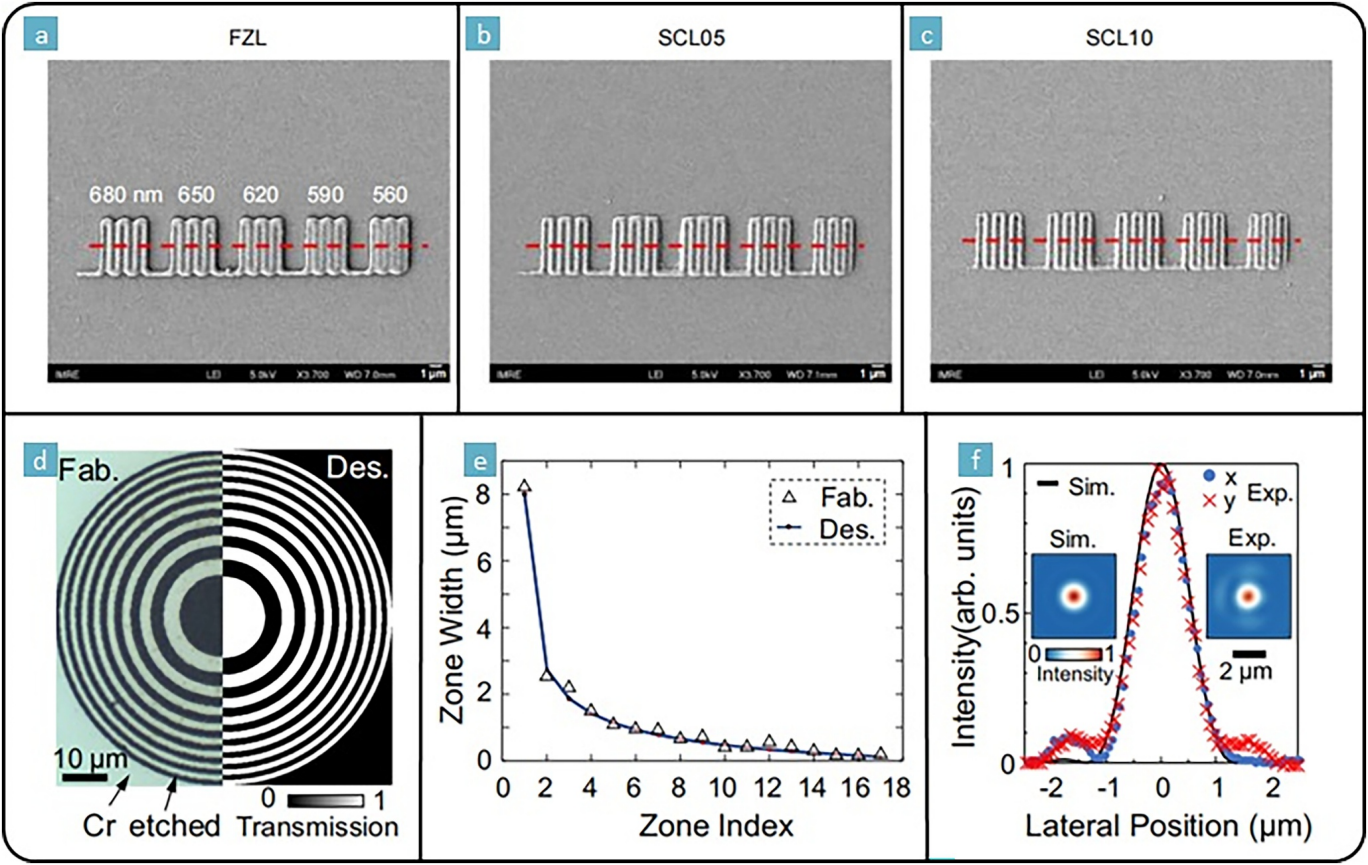
Subdiffraction‐limit lithography based on super‐oscillatory lenses. (a–c) SEM images of the grating patterns, from left to right, with pitches of 680, 650, 620, 590, and 560 nm and a length of 4 μm: (a) Grating written by a conventional Fresnel zone plate; (b) Grating written by an SCL with a sidelobe intensity ratio of 5%; (c) Grating written by an SCL with a sidelobe intensity ratio of 10%. Reproduced under the terms of the CC‐BY license [130]. Copyright 2024, the authors, published by the Institute of Optics and Electronics, Chinese Academy of Sciences. (d) Fabricated (left) and designed (right) binary‐amplitude zone plates with a focal length of 200 μm at a working wavelength of 405 nm. An image of the fabricated zone plates was obtained using reflective microscopy, revealing dark contrast in the etched region and bright contrast in the non‐etched Cr film. (e) Designed and measured zone widths of the FZP. (f) Simulated (solid curves) and measured (dots and forks) intensity profiles at the focal plane of the FZP. Insets: 2D intensity profiles of the focal spots. Reproduced under the terms of the CC‐BY license [133]. Copyright 2024, the authors, Springer Nature.

### 3.3 PSODLs for High‐Precision Measurement

PSODLs, demonstrating exceptional light‐field manipulation capabilities for both imaging and fabrication, offer several inherent advantages for high‐precision measurements. Subdiffraction‐limit focal spots can function as nanoscale rulers, enabling the measurement of parameters such as displacement and morphology. These capabilities enable new paradigms for multifunctional integrated measurements, enabling the simultaneous acquisition of information ranging from geometric dimensions to spectral data. This outcome represents the evolution of PSODLs from single‐function devices to intelligent measurement solutions defined by specific applications.

Conventional diffraction theory limits free‐space optical fields from forming subwavelength structures in the far field. Furthermore, traditional measurement techniques are constrained by the environmental sensitivity of interferometers, making stable operation in complex settings challenging. Nanoscale measurement based on subdiffraction‐limit focusing addresses these issues by utilizing the nanoscale localization characteristics of the light field as a probe or ruler [134]. Yuan et al. experimentally demonstrated that super‐oscillatory fields can generate extremely large local wave vectors, phase singularities, optical vortices, and regions of energy backflow, as shown in Figure 13a [135]. These features bear a high resemblance to plasmonic near‐fields. This work revealed that free‐space optical fields also possess nanoscale localization capability, establishing the physical foundation for their use as optical probes. Researchers have designed various super‐oscillatory focusing devices. For instance, Zhou et al. proposed a super‐oscillatory zoom metalens [136], which can switch between two focal lengths via polarization control, as illustrated in Figure 13b, enabling tunable nanoscale focusing. Liao et al. further developed a flat‐field super‐oscillatory lens [137], which maintains stable subwavelength focusing across a large FOV (9°), as shown in Figure 13c, making it suitable for scanning measurement systems.

**FIGURE 13:**
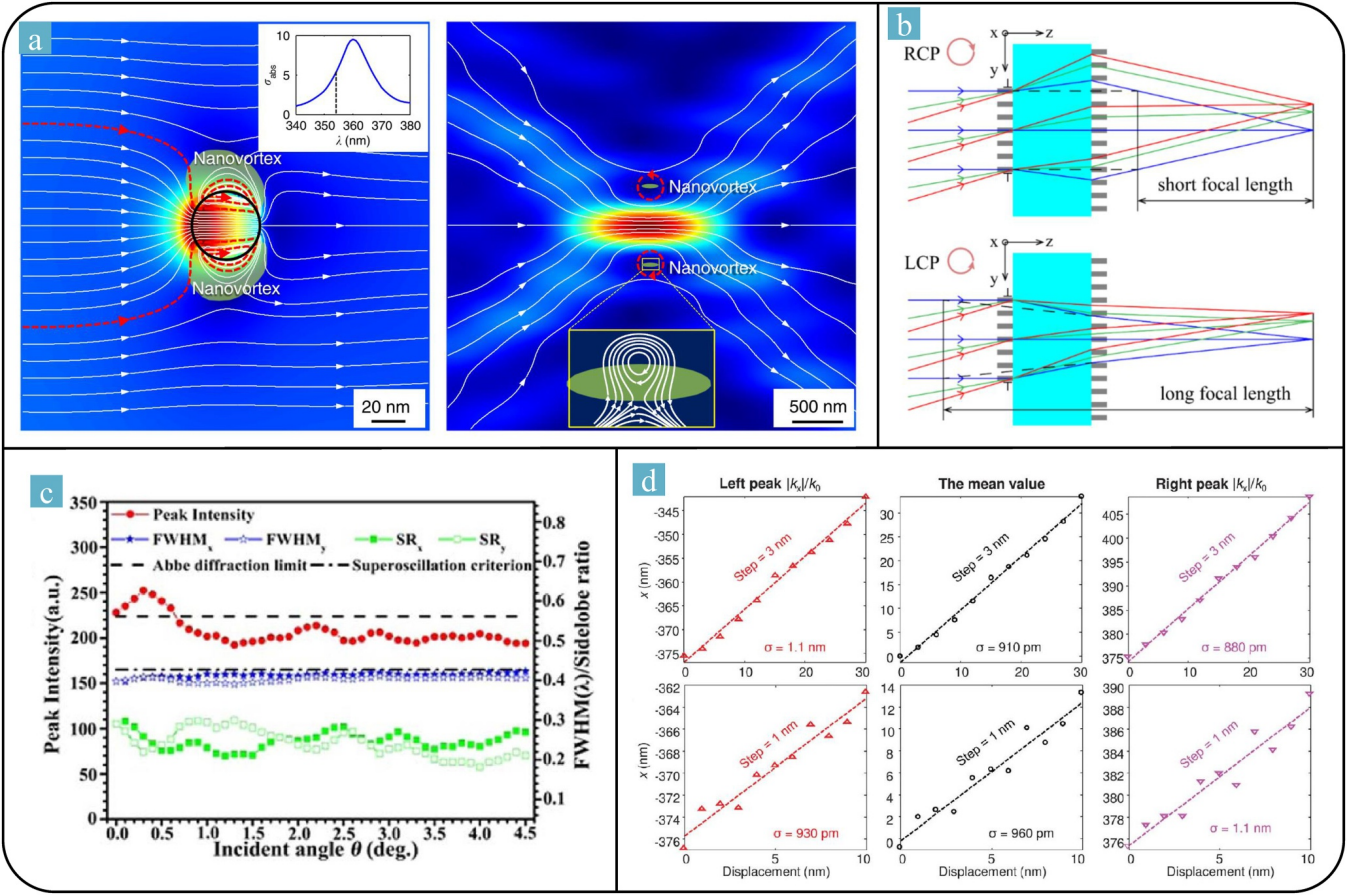
Development path of super‐oscillatory nanometrology. (a) Physical analogy: comparison of nanoscale focusing between a plasmonic nanoparticle (left) and a super‐oscillatory mask (right). Reproduced under the terms of the CC‐BY license [135]. Copyright 2019, the authors, Springer Nature. (b) Tunable device: schematic diagram of the polarization‐switchable, double‐layer step‐zoom metalens. Reproduced under the terms of the CC‐BY license [136]. Copyright 2022, the authors, published by the Beilstein‐Institut. (c) Focusing performance: key metrics (peak intensity, FWHM, SR) as functions of the incident angle. Reproduced with permission [137]. Copyright 2022, Chinese Laser Press. (d) Displacement measurement: experimental validation using the optical ruler with 3 nm (top) and 1 nm (bottom) steps, showing subnanometer precision (σ: standard deviation). Reproduced with permission [138]. Copyright 2019, published by the American Association for the Advancement of Science.

These devices serve as flexible and high‐performance “optical probes” for nanoscale measurements. Building on this capability, Yuan and Zheludev directly applied the nanoscale localization properties of super‐oscillatory fields to metrology, introducing the concept of an “optical ruler” [138]. By using phase singularities as scale marks, they experimentally demonstrated a displacement resolution better than 1 nm, as shown in Figure 13d. This breakthrough established a pathway for pushing the resolution of optical metrology to previously inaccessible scales. In 2024, Ma et al. proposed an infinitesimal optical singularity ruler using a transverse orbital angular momentum field, achieving three‐dimensional displacement measurement with picometer‐scale precision [139]. Compared to super‐resolution microscopy techniques such as STED and PALM/STORM [140, 141], the optical ruler achieves subnanometer displacement resolution without the need for fluorescence labeling, intense photobleaching, or lengthy data acquisition. This advancement marks the transition of super‐oscillatory focusing technology from proof‐of‐principle demonstrations to practical applications in nanoscale metrology.

### 3.4 Other PSODL Applications

This section explores extended applications of planar PSODLs in areas including data storage, optical sensing, and particle manipulation. These cases collectively demonstrate the potential of PSODLs, which extends beyond imaging and fabrication [142, 143, 144, 145, 146]. The ability of these devices to localize light fields at the subdiffraction limit, together with high flexibility in wavefront customization, supports applications that require higher efficiency, greater precision, and improved integration. Specifically, high‐density magnetic recording has been achieved using a far‐field super‐oscillatory focusing device capable of generating deep subwavelength hot spots in magnetic recording layers at 473 nm [147]. These focal spots exhibit long DOFs and can pass through solid immersion layers or air interfaces while locally heating the magnetic disk and enabling practical heat‐assisted magnetic recording applications, as shown in Figure 14a. Similarly, a novel 3D light‐induced magnetic holography technique has been proposed based on a conceptual supercritical design utilizing multibeam combinations in a 4π microscopic system. This approach employs 3D deep super‐resolved (purely longitudinal) magnetic hot spots to facilitate 3D magnetic holography with volumetric storage density [148], as shown in Figure 14b.

**FIGURE 14:**
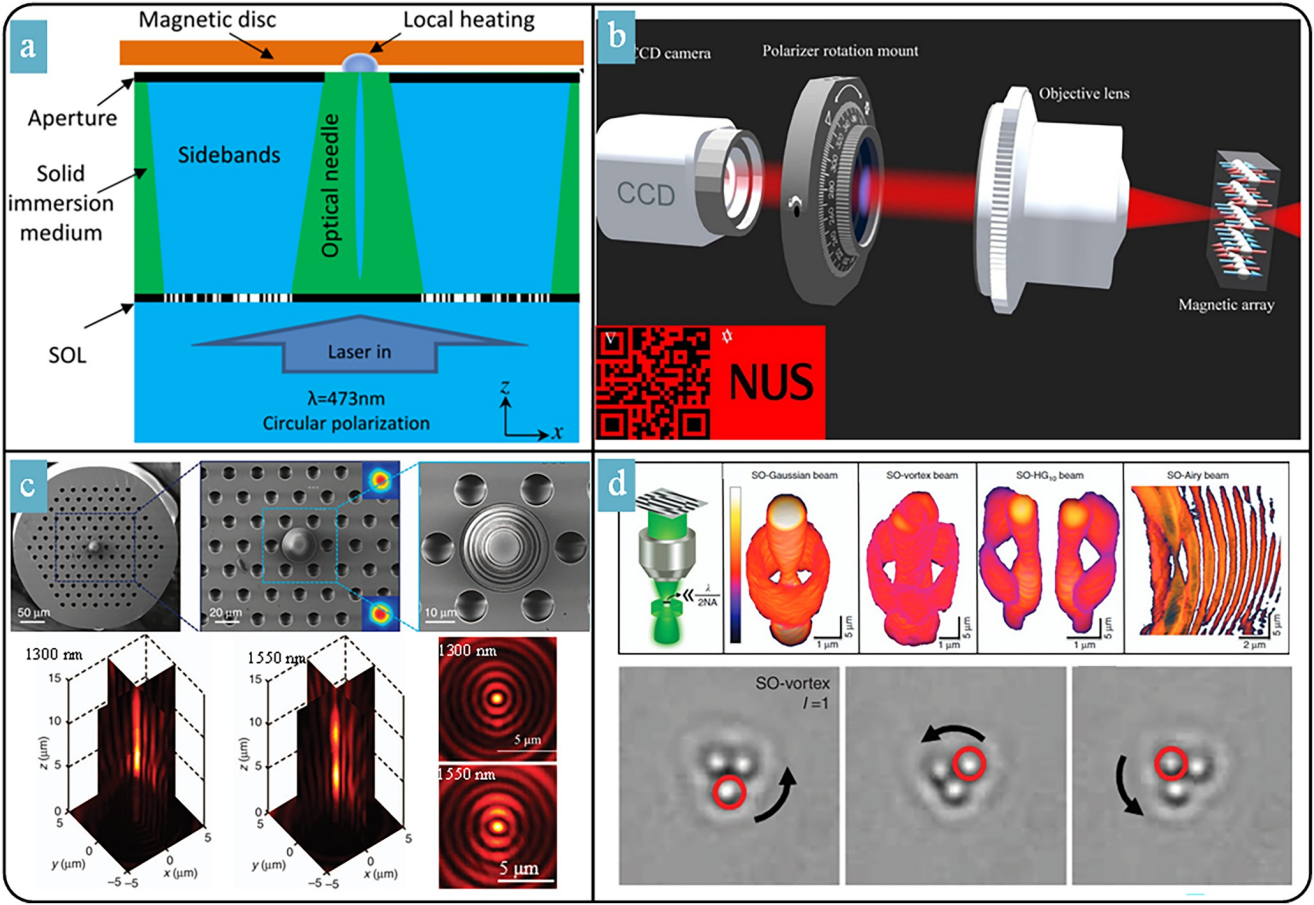
Other applications of SOLs. (a) Schematic configuration of an optical needle focused by an SOL in a solid immersion medium for heat‐assisted magnetic recording. Reproduced with permission [147]. Copyright 2014, Optical Society of America. (b) SOLs’ hot spot patterns are applied for identity verification. Reproduced under the terms of the CC‐BY license [148]. Copyright 2017, the authors, published by the American Association for the Advancement of Science. (c) Scanning electron microscope image and zoomed‐in views of the SOLs fabricated on the core of a single‐mode large‐mode‐area photonic crystal fiber. Reproduced under the terms of the CC‐BY license [72]. Copyright 2017, the authors, Springer Nature. (d) Generation of superoscillating beams. A time series showing the anticlockwise rotation of beads trapped in a super‐oscillatory vortex beam of charge *l* = 1 due to the transfer of orbital angular momentum *l* from the SO vortex beam to the trapped beads. Reproduced under the terms of the CC‐BY license [149]. Copyright 2017, the authors, Springer Nature.

In addition to high‐density data storage, fiberized SOL devices can function as optical modulation and shaping components in optical sensing systems [72], as shown in Figure 14c. A near‐infrared binary‐amplitude super‐oscillatory lens, implemented using a spatial light modulator, also provides a possible route for high‐precision optical trapping, nanoparticle manipulation, and structured beam generation for super‐resolution optical techniques. Structured super‐oscillatory beams can also be tuned to generate subdiffraction‐limit focal spots, helical phase fronts, or Airy‐like shapes [149]. These beams can then be used for the precise localization and trapping of nanoparticles. By using super‐oscillatory beams with opposite topological charges, nanoparticles can be rotated clockwise or counterclockwise, with a threefold improvement in localization precision and a 9.4‐fold enhancement in trapping stiffness, as illustrated in Figure 14d.

## 4 Discussion

Over the past decade, PSODLs, including SOLs and SCLs, have advanced rapidly in far‐field subdiffraction focusing, super‐resolution imaging, vectorial‐field control, broadband focusing, quasi‐nondiffracting beam generation, and terahertz industrial inspection [150, 151, 152, 153]. Unlike near‐field super‐resolution schemes that rely on the recovery of evanescent waves, PSODLs mainly generate subdiffraction localized optical fields in the far field through the coherent interference of propagating waves. However, their transition from laboratory demonstrations to practical applications remains constrained by several coupled challenges, including the trade‐offs among resolution, focusing efficiency, sidelobes, FOV, DOF, working bandwidth, fabrication tolerance, and system integration complexity [53]. Accordingly, and consistent with the application‐oriented scope of this review, future development of PSODLs should focus on the following key aspects.

### 4.1 Fundamental Performance Trade‐offs

PSODLs can generate subdiffraction focal spots in the far field, but this capability is usually accompanied by a redistribution of optical energy from the main lobe to sidelobes and background regions. As the FWHM is further reduced, the effective focusing efficiency tends to decrease, and background noise, artifacts, or unwanted energy deposition may arise in imaging, detection, or laser processing [51]. Therefore, the performance of PSODLs should not be evaluated only by focal‐spot size but also by the main‐lobe energy fraction, sidelobe intensity, focusing efficiency, signal‐to‐noise ratio, and image contrast. In addition, high resolution, large FOV, long DOF, and broadband operation or achromatic response are mutually coupled. Focal shifts and aberrations under different wavelengths, off‐axis illumination, or thick‐sample conditions can further affect practical performance. Future designs should, therefore, move from single‐metric optimization toward application‐oriented multiobjective optimization and trade‐off management.

### 4.2 Inverse Design and Algorithm Selection

To manage the trade‐offs outlined above, inverse design, topology optimization, and deep learning approaches can be used to explore complex PSODL parameter spaces and optimize coupled metrics such as focal‐spot size, focusing efficiency, sidelobe level, and polarization response [154, 155, 156]. Meanwhile, microelectromechanical systems, tunable materials, and active optical platforms may enable dynamic focusing, FOV scanning, and beam control in PSODLs [157, 158, 159]. Nevertheless, these methods also introduce new issues, such as algorithm generalization, packaging and alignment, driving stability, response speed, power consumption, and system loss.

### 4.3 Manufacturability and System Reliability

The subdiffraction focal fields of PSODLs rely on accurate amplitude or phase distributions over the entire aperture. As a result, dimensional deviations, edge roughness, film‐thickness fluctuations, material‐index variations, and alignment errors may lead to focal‐spot broadening, enhanced sidelobes, reduced efficiency, or focal shifts. These errors can directly affect imaging contrast, sensing repeatability, and the stability of energy deposition in optical storage or laser processing. Therefore, manufacturability and error tolerance are not only fabrication concerns but also practical constraints for the deployment of PSODLs. Future work should incorporate fabrication constraints and error models at the design stage while developing scalable, low‐cost fabrication methods with high process consistency, together with reliable validation protocols. Large‐scale direct laser writing of high‐NA SOLs and related 3D‐printed terahertz metalenses illustrates possible fabrication routes, although systematic tolerance and batch‐consistency studies of PSODLs are still needed [160, 161].

### 4.4 Standardized Evaluation and Application Validation

PSODLs have shown potential in super‐resolution imaging, precision measurement, beam shaping, and terahertz industrial inspection, whereas related planar optical‐field‐control devices are also being extended to atom manipulation and high‐density optical storage [162, 163]. However, the requirements vary across applications. Imaging systems prioritize resolution, sidelobe noise, and image contrast; sensing and measurement systems pay more attention to focus stability, signal‐to‐noise ratio, and environmental adaptability, whereas optical storage and laser processing systems are more concerned with energy concentration, threshold control, and repeatability. Currently, the definitions of FWHM, focusing efficiency, SR, effective FOV, DOF, and working bandwidth remain inconsistent across studies, limiting fair comparison among designs. Future studies should adopt more unified evaluation metrics and verify device performance in realistic application scenarios rather than only demonstrating a single subdiffraction focal spot under ideal conditions.

### 4.5 System‐Level Integration and Emerging Applications

Beyond their role as stand‐alone focusing elements, PSODLs may be integrated with light sources, scanning modules, detectors, and computational reconstruction algorithms in compact optical systems. Such combinations could support miniature imaging, adaptive inspection, and chip‐scale sensing platforms; a terahertz‐wave cylindrical SOL has already been investigated for industrial applications [153]. Water‐immersion SCLs have demonstrated low‐sidelobe far‐field super‐resolution focusing in liquid environments, providing a potential route toward label‐free microscopy and other liquid‐compatible systems [164]. Devices designed for reproducible spatial energy delivery could be applied to laser nanofabrication and multilayer optical storage, whereas related super‐oscillatory‐light approaches have demonstrated single‐shot 2D nanometrology and may inform future PSODL‐based measurement systems [165]. Broadband or dispersion‐engineered designs may further enable compact spectral sensing and wavelength‐multiplexed optical processing. The value of these prospective applications should be established through task‐level outcomes, such as pattern fidelity, detection sensitivity, trapping stability, throughput, instrument compactness, and long‐term operational stability under realistic operating conditions rather than focal‐spot characteristics alone.

## 5 Conclusion

Planar optical devices capable of far‐field subdiffraction optical‐field manipulation are gradually moving from proof‐of‐concept demonstrations toward application‐oriented device development. In this review, we summarize recent progress in the development and application of PSODLs within a unified PSODL framework that includes SOLs and SCLs. By comparing the performance requirements of different application scenarios, this review focuses on the design strategies and application potential of PSODLs in super‐resolution imaging, micro/nanofabrication, and precision measurement while further discussing their extensions to particle manipulation, optical storage, and optical sensing. Nevertheless, further development of PSODLs is still constrained by multiple trade‐offs among resolution, focusing efficiency, sidelobe suppression, FOV, DOF, working bandwidth, fabrication tolerance, and system reliability. Therefore, future studies need to move beyond the demonstration of single focal‐spot performance toward application‐oriented multiobjective optimization, manufacturability‐aware design, active system integration, and standardized evaluation. By linking fundamental physical limitations, device design strategies, and practical application requirements, PSODLs may become key components of next‐generation compact flat optical systems.

## Author Contributions

**Yutian Cheng:** conceptualization, methodology, writing – original draft, writing – review and editing, resources. **Lingyi Tang:** writing – review and editing. **Xingcheng Xiao:** funding acquisition, writing – review and editing, project administration. **Xiaochang Yu:** funding acquisition, writing – review and editing, project administration. **Ya Guo:** writing – review and editing. **Yiting Yu:** writing – review and editing, funding acquisition. **Wenli Li:** writing – original draft, writing – review and editing, conceptualization, funding acquisition, methodology.

## Data Availability

The data that support the findings of this study are available from the corresponding author upon reasonable request.
